# Bridging the gap between HIV epidemiology and antiretroviral resistance evolution: Modelling the spread of resistance in South Africa

**DOI:** 10.1371/journal.pcbi.1007083

**Published:** 2019-06-24

**Authors:** Anthony Hauser, Katharina Kusejko, Leigh F. Johnson, Gilles Wandeler, Julien Riou, Fardo Goldstein, Matthias Egger, Roger D. Kouyos

**Affiliations:** 1 Institute of Social and Preventive Medicine, University of Bern, Switzerland; 2 Division of Infectious Diseases and Hospital Epidemiology, University Hospital Zurich, University of Zurich, Zurich, Switzerland; 3 Institute of Medical Virology, University of Zurich, Zurich, Switzerland; 4 Centre for Infectious Disease Epidemiology and Research, University of Cape Town, South Africa; 5 Department of Infectious Diseases, Bern University Hospital, University of Bern, Bern, Switzerland; Bordeaux Population Health, FRANCE

## Abstract

The scale-up of antiretroviral therapy (ART) in South Africa substantially reduced AIDS-related deaths and new HIV infections. However, its success is threatened by the emergence of resistance to non-nucleoside reverse-transcriptase inhibitors (NNRTI). The MARISA (Modelling Antiretroviral drug Resistance In South Africa) model presented here aims at investigating the time trends and factors driving NNRTI resistance in South Africa. MARISA is a compartmental model that includes the key aspects of the local HIV epidemic: continuum of care, disease progression, and gender. The dynamics of NNRTI resistance emergence and transmission are then added to this framework. Model parameters are informed using data from HIV cohorts participating in the International epidemiology Databases to Evaluate AIDS (IeDEA) and literature estimates, or fitted to UNAIDS estimates. Using this novel approach of triangulating clinical and resistance data from various sources, MARISA reproduces the time trends of HIV in South Africa in 2005–2016, with a decrease in new infections, undiagnosed individuals, and AIDS-related deaths. MARISA captures the dynamics of the spread of NNRTI resistance: high levels of acquired drug resistance (ADR, in 83% of first-line treatment failures in 2016), and increasing transmitted drug resistance (TDR, in 8.1% of ART initiators in 2016). Simulation of counter-factual scenarios reflecting alternative public health policies shows that increasing treatment coverage would have resulted in fewer new infections and deaths, at the cost of higher TDR (11.6% in 2016 for doubling the treatment rate). Conversely, improving switching to second-line treatment would have led to lower TDR (6.5% in 2016 for doubling the switching rate) and fewer new infections and deaths. Implementing drug resistance testing would have had little impact. The rapid ART scale-up and inadequate switching to second-line treatment were the key drivers of the spread of NNRTI resistance in South Africa. However, even though some interventions could have substantially reduced the level of NNRTI resistance, no policy including NNRTI-based first line regimens could have prevented this spread. Thus, by combining epidemiological data on HIV in South Africa with biological data on resistance evolution, our modelling approach identified key factors driving NNRTI resistance, highlighting the need of alternative first-line regimens.

## Author summary

Resistance to non-nucleoside reverse transcriptase inhibitors (NNRTI) threatens the long-term success of antiretroviral therapy (ART) roll-out in South Africa. We developed a compartmental model integrating the local HIV epidemiology with biological mechanisms of drug resistance. A first dimension of the model accounts for the continuum of care: infection, diagnosis, first-line treatment with suppression or failure, and second-line treatment. Other dimensions include: disease progression (CD4 counts), gender, and acquisition and transmission of NNRTI resistance. Whenever possible, we informed the parameters using the data available from local cohorts. Other parameters were informed using literature or UNAIDS estimates. The model captured the rise of NNRTI resistance during the period. We assessed the impact of counter-factual scenarios reflecting alternative countrywide policies during the period 2005 to 2016, considering either increasing ART coverage, improving management of treatment failure, broadening ART eligibility, or implementing drug resistance testing before ART initiation. We identified key drivers of the NNRTI resistance epidemic: large-scale ART roll-out and insufficient monitoring of first-line treatment failure. The model also suggested that no policy including NNRTI-based first line regimens could have prevented the spread of NNRTI resistance.

## Introduction

Since ART has been introduced in Southern Africa in 2004, ART coverage has continuously increased. In 2016, 55% of individuals living with HIV were receiving ART in the region, the great majority being treated with a standard first-line regimen consisting of two nucleoside reverse transcriptase inhibitors (NRTI) and one non-nucleoside reverse transcriptase inhibitor (NNRTI) [1]. The scale-up of ART led to a substantial reduction in mortality but the emergence of drug resistance could jeopardize its long-term success [2]. Of particular concern are NNRTIs, as this class has a relatively low genetic barrier to resistance [3]. As documented by the World Health Organization (WHO), the level of pretreatment NNRTI resistance has rapidly increased and reached the 10% threshold in the Southern Africa region in 2015 [4]. According to WHO, this threshold should trigger considerations on changing the first-line regimen. By contrast, resistance to NRTIs, though relevant at the individual level, is only rarely transmitted [4].

In South Africa, adult HIV deaths have decreased from 220,000 in 2006 to 99,000 in 2014 [2]. In 2016, an estimated 63% of HIV positive people were on ART in South Africa [1]. While initially only people with CD4 counts lower than 200 cells/μL were eligible to start ART, South Africa adopted the “Treat All” policy in 2017, which recommends ART for all HIV-positive people regardless of their CD4 counts [5]. The goal is to reach 90% of diagnosed people on ART in 2020, in line with the 90-90-90 targets of UNAIDS [6]. While the HIV epidemic in South Africa has been well described and extensively modelled [2,7,8], relatively little work has been done on drug resistance [9,10]. The rapid increase in ART coverage might fuel further increase in drug resistance as more and more people become exposed to the drug, but the impact of the scaling up of ART on the development of NNRTI resistance is not well defined at present. Another key question is whether a better management of treatment failure would have mitigated NNRTI resistance.

While understanding the drivers of antiretroviral resistance is crucial for public health, representative, longitudinal data on drug resistance are scarce, compared to the large amount of cohort data available on the clinical and public health epidemiology of HIV. Moreover, quantifying the spread of resistance is challenging because it involves both epidemiological (transmission, cascade of care, disease progression) and evolutionary processes (emergence and selection of resistance mutations) [11–13], with the parameters governing the latter typically unknown [13].

We aimed to capture the dynamics of NNRTI resistance in South Africa during 2005–2016 and to quantify the impact that different policy changes would have had on the rise of drug resistance. To this end we developed MARISA (Modelling Antiretroviral drug Resistance In South Africa), a mathematical model integrating the specificities of HIV epidemiology in the country with the evolutionary epidemiology of drug resistance. MARISA is a compartmental, deterministic model whose structure reflects gender-specific dynamics of continuum of care and disease progression, as well as acquisition and transmission of HIV NNRTI resistance. We calibrated the model using data from the International epidemiology Databases to Evaluate AIDS in Southern Africa (IeDEA-SA, www.iedea-sa.org, [14]), literature estimates and HIV key outcomes provided by UNAIDS [1]. The acquisition and transmission of NNRTI drug resistance was integrated within the general dynamics of the HIV epidemic in the country and parametrized with estimates derived from other cohorts. This allowed the estimation of the yearly levels of acquired and transmitted drug resistance (ADR and TDR, respectively). We then assessed the impact of counter-factual scenarios reflecting alternative countrywide public health policies, including policies of increasing ART coverage, improving management of treatment failure, broadening ART indications, or implementing drug resistance testing before initiation.

## Method

### Model structure

MARISA is a mechanistic, compartmental model. The first dimension of the model accounts for the whole continuum of care: infection of susceptible individuals, diagnosis, first-line treatment including NNRTI with subsequent suppression or failure, and second-line treatment including protease inhibitors (PI) with subsequent suppression or failure (8 classes). We then consider three additional dimensions: disease progression as characterized by CD4+ T cell counts (4 classes); NNRTI resistance status (2 classes); and gender (2 classes). This leads to a total of 128 compartments. The first two dimensions describe the different care stages and their interaction with HIV progression. The third dimension is key to capture the acquisition of NNRTI resistance by individuals with first-line treatment failure (with rate *σ*_*res*_), the transmission of resistant strains of HIV to susceptible individuals, and the reversion of HIV resistance mutations when no more drug pressure is exerted (with rate *σ*_*rev*_). We assume that individuals infected with the NNRTI resistant virus have higher failure and lower viral suppression rates (hazard ratio *α* and *α*^−1^, respectively). As one mutation (e.g. the K103N mutation) alone confers high-level resistance to NNRTI drugs [15], only one layer is used to represent NNRTI resistance. The fourth dimension reflects differences observed between women and men, with diagnosis and treatment rates being higher for women than for men [16,17]. This dimension is also involved in modelling HIV transmission among adults (≥15 years old).

**Fig 1:**
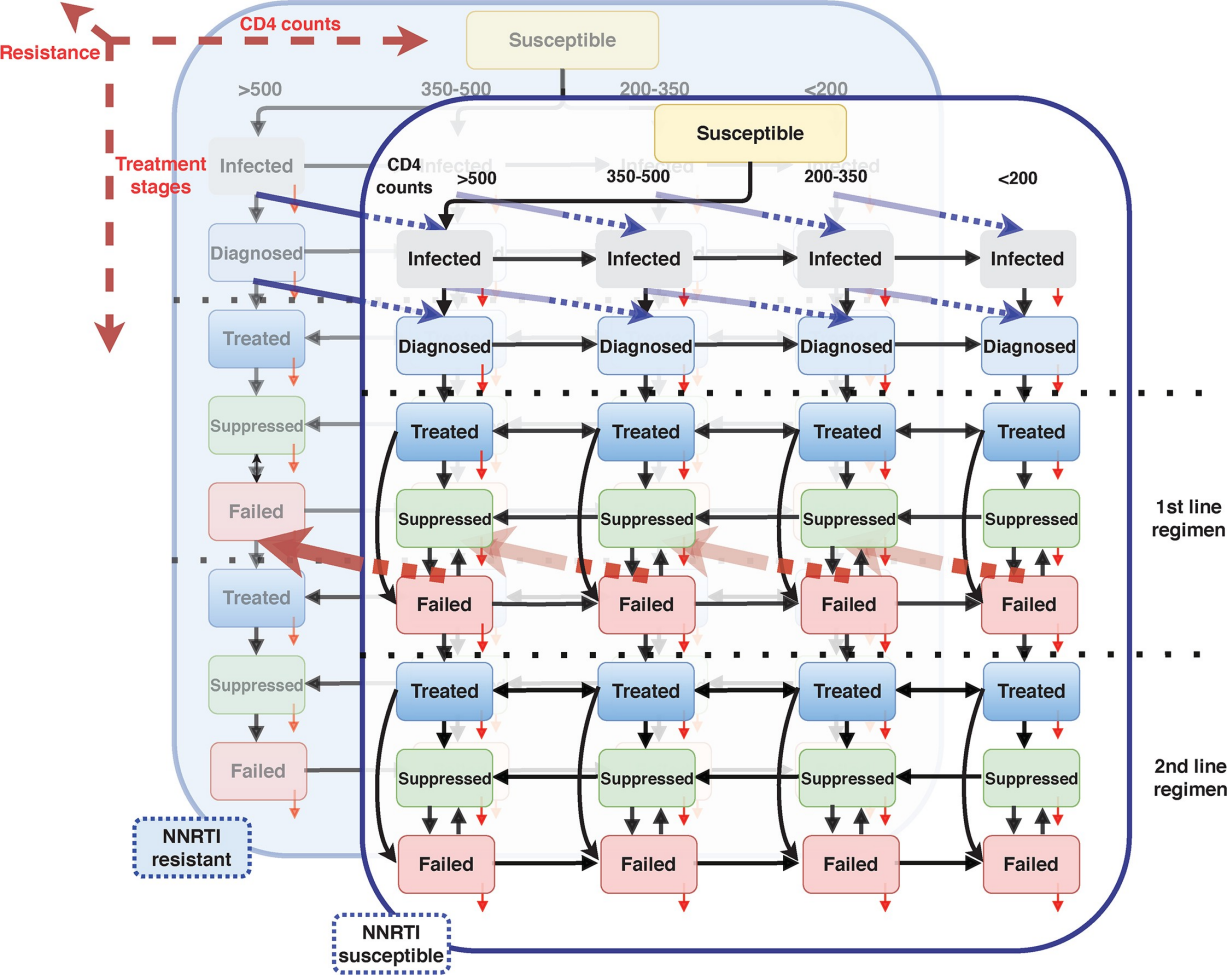
Compartmental model. Three of the four dimensions are represented: 1) care stages (vertically), 2) disease progression (horizontally, stratified in 4 CD4 counts strata), 3) NNRTI resistance (represented by the two overlapped layers). For sake of clarity, arrows representing treatment interruption are not displayed. Red arrows represent acquisition of NNRTI resistance, while blue arrows represent reversion to wild-type HIV-strain.

Movement between compartments is determined by different rates, some of which change over time to reflect modifications in treatment policies or in behavior. Adults living in South Africa who are not infected are represented by the susceptible compartment (*Susc*), as shown in Fig 1. The *I* compartments represent undiagnosed HIV-positive individuals. The force of infection considers three transmission routes among adults: a man can either be infected by a woman (“heterosexual” or HET transmission) or, less commonly, by a man (“men having sex with men” or MSM transmission), while a woman can only be infected by a man. HET and MSM populations are only implicitly modelled: we assume a density-dependent transmission that accounts for different risk behaviors according to knowledge of HIV status (monthly number of unprotected sexual contacts *β*_*u*_ and *β*_*d*_ for undiagnosed and diagnosed HIV-infected individuals, respectively) and the expected proportion of HET and MSM among men. Inflow of infected children reaching the age of 15 is also taken into account by using estimates from the Thembisa model and published literature (See Section 1.5 in S1 File)[2,17–19]. Infected individuals become diagnosed at a rate *γ*_*I*→*D*_(*t*) that is allowed to vary over time, by CD4 count and by gender. Once diagnosed (compartment *D*), individuals will start treatment at a rate $\gamma_{D\to T_{1}}(t)$ that also varies over time, reflecting the successive changes in ART guidelines. This rate also depends on the CD4 count, as individuals with lower counts will initiate treatment at higher rates (see Section 1.3 in S1 File).

First-line ART initiation is represented by the *T*_*1*_ compartment, which characterizes individuals who have been on ART for three months or less. After this period, they can either suppress viral replication (*S*_*1*_) or fail treatment (*F*_*1*_). These two compartments reflect the use of viral load monitoring in South Africa to identify patients failing first-line treatment that should switch to second-line regimen. We assume that virally suppressed individuals cannot transmit the virus. When failing first-line treatment, individuals are switched to second-line treatment (compartment *T*_*2*_) at rate $\gamma_{F_{1}\to T_{2}}$. Care and disease progression on second line treatment are modelled identically to first-line therapy. Mortality at each stage differs according to disease progression and care stage. In addition, the mortality rates for patients with CD4 counts below 200 cells/μl are time-dependent, due to the highly variable mortality risk in this class [20]. Overall, the model contains 137 different rates. The total population of each gender follows the WHO estimates for South Africa, and initial conditions in each compartment reflect UNAIDS estimates for 2005. Further details on the MARISA model are available in Sections 1 and 2 in S1 File.

### Parameter values and calibration procedure

We parameterized and calibrated the model in two successive steps. First, some parameters were given fixed values using external sources. Literature estimates were used for parameters related to NNRTI resistance (*σ*_*res*_, *σ*_*rev*_, and *α*), for transmission probabilities per sexual contact, for the proportion of MSM and for the mortality risks (relatively to suppressed individuals with more than 500 CD4/μL). Similarly, values were defined for the time-dependent diagnosis rates (differentiating between testing asymptomatic individuals, symptomatic individuals and pregnant women, and relatively to the treatment rate in 2005) and treatment rates (relatively to the treatment rate for an eligible individual with less than 200 CD4/μL in 2005). We used estimates from studies conducted in South Africa whenever available. For parameters related to disease progression (movements between CD4 strata) and to the continuum of care after starting first-line treatment (rates of suppression, treatment failure, switching to second line, and treatment interruption), we used data from five IeDEA cohorts in South Africa (Aurum Institute, Hlabisa, Khayelitsha, Kheth’Impilo and Tygerberg) that provided longitudinal information for 54,016 HIV-infected adults [14]. The majority of them were female (62%). All patients started a first-line regimen and 3905 (7.2%) received a second-line regimen. Viral load measurements were used to identify the occurrence of suppression or treatment failure in treated individuals (using a threshold of 1000 copies/mL). Because of low monitoring frequency, the number of available measurements per patient was limited and some intermediate steps in disease or care progression were missing. We thus adapted methods from survival analysis in order to reconstruct patients’ care histories (see Section 3.1 in S1 File). See Table 1 for more details about parameters.

**Table 1:** Parameters used in the model. **IeDEA cohort data were used to estimate clinical progression rates.** Parameters that could not be estimated with these data were collected from literature. Finally, time-varying parameters were estimated by fitting the MARISA model to estimates from Thembisa model.

Parameters	Definition	Reference
**Parameters obtained from literature (see Table 4 in S1 File)**		
*Resistance parameters*		
*σ*_*res*_	Rate of acquiring NNRTI resistance when failing 1^st^-line treatment	[21]
*σ*_*rev*_	Reversion rate when no more NNRTI-drug pressure	[22]
*α*	Positive impact of NNRTI resistance on treatment failure	[23]
*HIV-transmission parameters*		
	Probabilities of HIV infection across gender	[24]
	MSM prevalence	[25]
*Mortality parameters*		
	Relative mortality risks across CD4 strata and care treatment status	[20,26]
*Diagnosis and treatment rates*		
*γ*_*I*→*D*_(*t*) $\gamma_{D\to T_{1}}(t)$	Diagnosis rates according to gender and CD4 strata Treatment rates according to CD4 strata	[17] [17]
**Parameters estimated of IeDEA cohort data by survival analysis (see Tables 2 and 3 in S1 File)**		
*Parameters related to disease progression*		[14]
	*Transition rates between CD4 strata*	
*Parameters related to continuum of care*		
$\gamma_{T_{1}\to S_{1}},\;\gamma_{F_{1}\to S_{1}},\;\gamma_{T_{2}\to S_{2}},\;\gamma_{F_{2}\to S_{2}}$	Suppression rates for first- and second-line treatment	
$\gamma_{T_{1}\to F_{1}},\;\gamma_{S_{1}\to F_{1}},\;\gamma_{T_{2}\to F_{2}},\;\gamma_{S_{2}\to F_{2}}$	Failure rates for first- and second-line treatment	
$\gamma_{F_{1}\to T_{2}}$	Switching rate from first- to second-line treatment	
$\gamma_{T_{1}\to D},\;\gamma_{S_{1}\to D},\;\gamma_{F_{1}\to D},\;\gamma_{T_{2}\to D},\;\gamma_{S_{2}\to D},\;\gamma_{F_{2}\to D}$	Treatment interruption rates	
**Parameters estimated by fitting MARISA to Thembisa model data (see Table 5 in S1 File)**		
*β*_*u*_, *β*_*d*_	monthly numbers of unprotected sexual contacts for undiagnosed and diagnosed people respectively	[1,17]
*γ*_*I*→*D*_(2005), *γ*_*I*→*D*_(2016)/*γ*_*I*→*D*_(2005)	base diagnosis rate in 2005 and its increase between 2005 and 2016	
$\gamma_{D\to T_{1}}(2005)$	treatment rate in 2005	
*q*	scale parameter modelling the decrease in the proportion of individuals with CD4<50 cells/μL	
$\mu_{S1/s2}^{i=1}$	mortality rate of suppressed individuals with >500 CD4/μL.	

**Fig 2:**
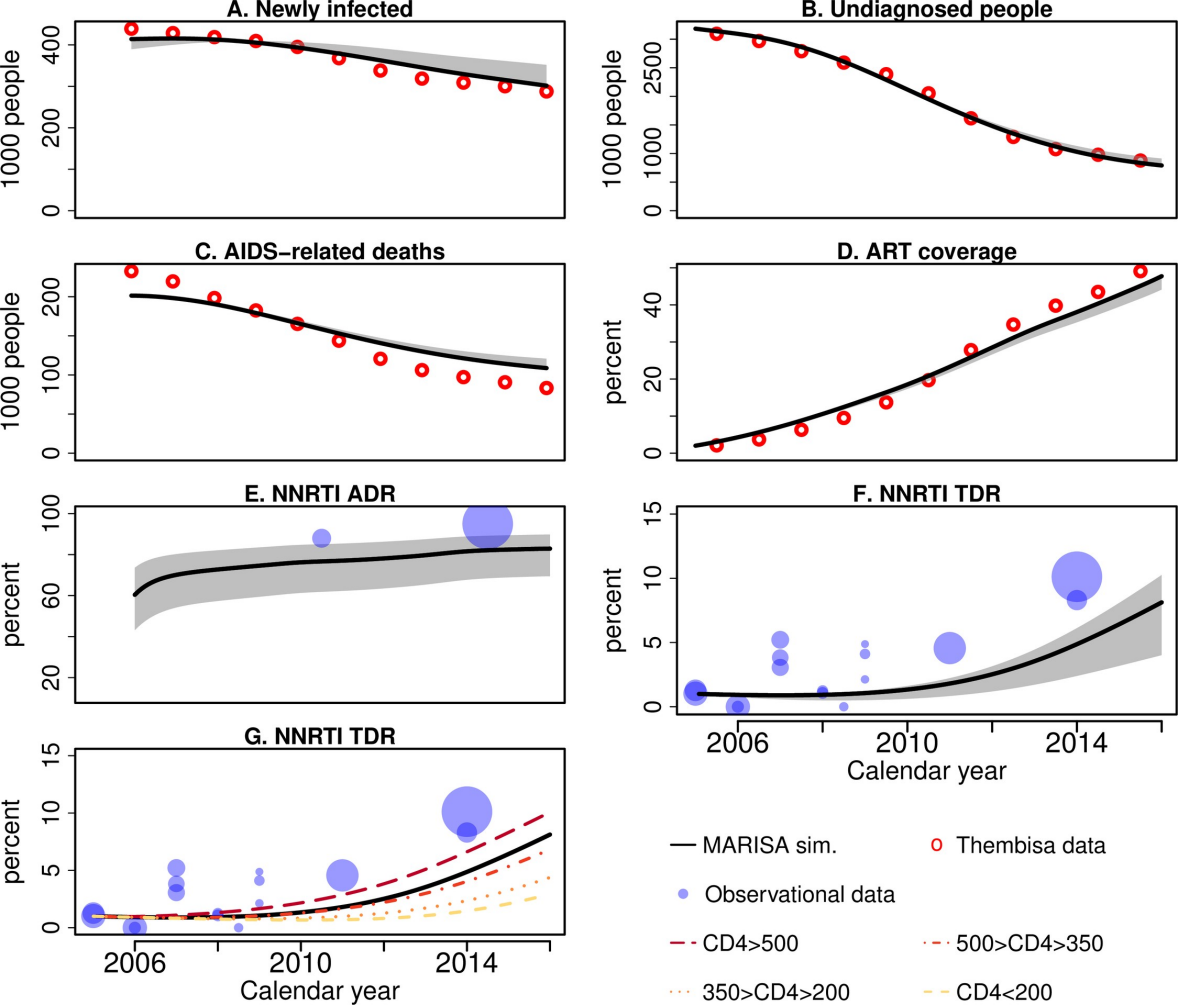
Best fit of the model. The plots a, b, c and d correspond to the four outcomes used during the fitting procedure: A) the number of newly infected per year, B) the total number of undiagnosed individuals at each year, C) the number of AIDS-related deaths per year and D) the percentage of infected individuals that are on ART. NNRTI ADR and TDR levels are displayed in E and, F and G respectively, and are not used to fit the model. Lines correspond to model output and circles to Thembisa estimates (in red) or to results from cross-sectional studies (in blue, see Table 6 in S1 File). Grey shades correspond to 100% sensitivity ranges. See Table 2 for more details.

During the second phase, the 7 remaining unknown parameters were estimated by fitting the model to estimates from the Thembisa model for the period 2005 to 2015: annual numbers of new HIV infections, number of undiagnosed individuals, annual number of AIDS-related deaths and ART coverage (Table 2 and Fig 2). The Thembisa model is a compartmental model providing UNAIDS with estimates on the South African HIV epidemic. Inference relied upon a maximum likelihood approach, assuming Poisson-distributed errors. We thus obtained point estimates for the monthly numbers of unprotected sexual contacts *β*_*u*_ and *β*_*d*_, for the base diagnosis rate in 2005 and its increase between 2005 and 2016, for the treatment rate in 2005, for a scale parameter modelling the decrease in the proportion of individuals with CD4 <50 cells/μL (only used for mortality estimates), and for the mortality rate of suppressed individuals with more than 500 CD4/μL (see Table 1). Further details are available in the Section 3 in S1 File.

**Table 2:** Outcomes and data sources used to calibrate the model and to compare the resistance related outcomes of the model. The six outcomes are displayed in Fig 2. See Section 3.2 in S1 File for more details.

Outcome	Definition	Source	Reference
**Data used during the fitting procedure**			
*New infections*	Number of newly HIV-infected adults per year	Thembisa model	[17]
*Undiagnosed people*	Number of undiagnosed HIV-infected adults	Thembisa model	[17]
*AIDS-related deaths*	Number of AIDS-related deaths per year (for adults)	Thembisa model	[17]
*Treatment coverage*	Percentage of HIV-infected adults that are treated	UNAIDS data	[1]
**Resistance estimates from cross-sectional studies**			
*Level of NNRTI ADR*	Percentage of people failing first-line treatment that are resistant to NNRTI	2 cross-sectional studies done in 2010 and 2014 in South Africa	[21,27]
*Level of NNRTI TDR*	Percentage of treatment-naïve people that are resistant to NNRTI	Data from a systematic review on the prevalence of PDR in South Africa, among other low and middle income countries.	[28]

### Simulations and counterfactual scenarios

The model was simulated from 2005 to 2016 using the specified parameter values and a monthly time step. Several outcomes were computed from the output, including the proportions of NNRTI ADR (proportion of individuals in *F*_*1*_ compartments with NNRTI resistance, see Eq 15 in S1 File) and of NNRTI TDR (proportion of individuals coming from *I* to *D* compartments with NNRTI resistance). When not specified otherwise, NNRTI TDR is measured in newly diagnosed patients (D) (see Eq 16 in S1 File). Alternatively, we determine the proportion of NNRTI resistance in newly infected patients, newly diagnosed patients or in ART initiators. In this latter case, as it comprises drug-experienced people, we used the term pre-treatment drug resistance (PDR), rather than TDR.

Four counterfactual scenarios were examined with the model. The first counterfactual scenario assessed the impact of treatment initiation ($\gamma_{D\to T_{1}}$) increased by factors 2, 3 or 5. The second counterfactual scenario investigated the impact of an earlier switch to second-line regimen ($\gamma_{F_{1}\to T_{2}}$) when failing the first-line regimen, by factors 2, 5 or 10. The third and fourth scenario examined the impact of different testing and treatment policies. In the third scenario, the “Treat All” policy, i.e. initiating first-line treatment of diagnosed individuals regardless of CD4 counts, was implemented at a hypothetical earlier point in time (moved forward by 1.5, 3 or 6 years). The fourth scenario implemented drug resistance testing and immediate second-line treatment of individuals harboring a resistant strain at baseline.

### Sensitivity analysis

We performed a multivariate sensitivity analysis in order to quantify the impact of uncertainty on the values of 1) four parameters related to NNRTI resistance (*σ*_*res*_, *σ*_*rev*_, *α* and the rates of treatment interruption) and 2) three parameters related to HIV transmission (percentage of MSM, probability of male-to-male infection per sexual contact, and ratio between HIV prevalence in MSM and HET). Multivariate uncertainty within specified ranges was introduced using Latin hypercube sampling [29]. Each model estimate is reported with a 100% sensitivity range. Further details are available in Section 4.2 in S1 File.

## Results

### Model outcomes

The model reproduces the main time trends of the HIV epidemic in South Africa 2005–2016 (Fig 2A–2D). There is a clear increase in ART coverage since 2005, attaining 48% of infected individuals in 2015, and a significant drop in the number of undiagnosed individuals, as a result of the increasing number of HIV tests performed annually. In 2015, the model estimated that 0.79 million of the 6.9 million infected individuals (11.4%) were not yet diagnosed. The number of yearly newly-infected individuals decreased from over 400,000 individuals in 2006 to about 300,000 in 2016. The decrease in risk behavior due to testing among HIV-positive individuals is estimated at 46% (*β*_*d*_/*β*_*u*_ = 0.54), in line with a behavioral study conducted in South Africa in 2013 [30]. Finally, HIV-related deaths dropped from over 200,000 in 2006 to 109,000 in 2016.

The MARISA model also captures the dynamics of NNRTI ADR and TDR, showing very high levels of ADR (Fig 2E) and increasing levels of TDR (Fig 2F) in South Africa after 2004. The model estimates that 73% of the individuals failing the first-line regimen had ADR to NNRTI in 2008, with a slight yet steady increase in the following years, surpassing 83% in 2016. Moreover, the model estimated that 13.8% of these individuals were already resistant at the time of failure. NNRTI TDR among newly diagnosed individuals increased from 0.9% to 8.1% during the period. Interestingly, the model indicates substantial variation in TDR levels over the four CD4 strata, ranging from 2.9% for newly diagnosed individuals with less than 200 CD4/μL to 10.0% for those with more than 500 CD4/μL in 2016. For newly infected individuals, the NNRTI TDR level reaches 15.0% in 2016. We also observe a high PDR prevalence among individuals initiating first-line ART (6.5% in 2016). Finally, the model estimated that 16.9% of ADR cases in 2016 were related to TDR (see Eq 17, in S1 File).

### Counterfactual scenarios

**Fig 3:**
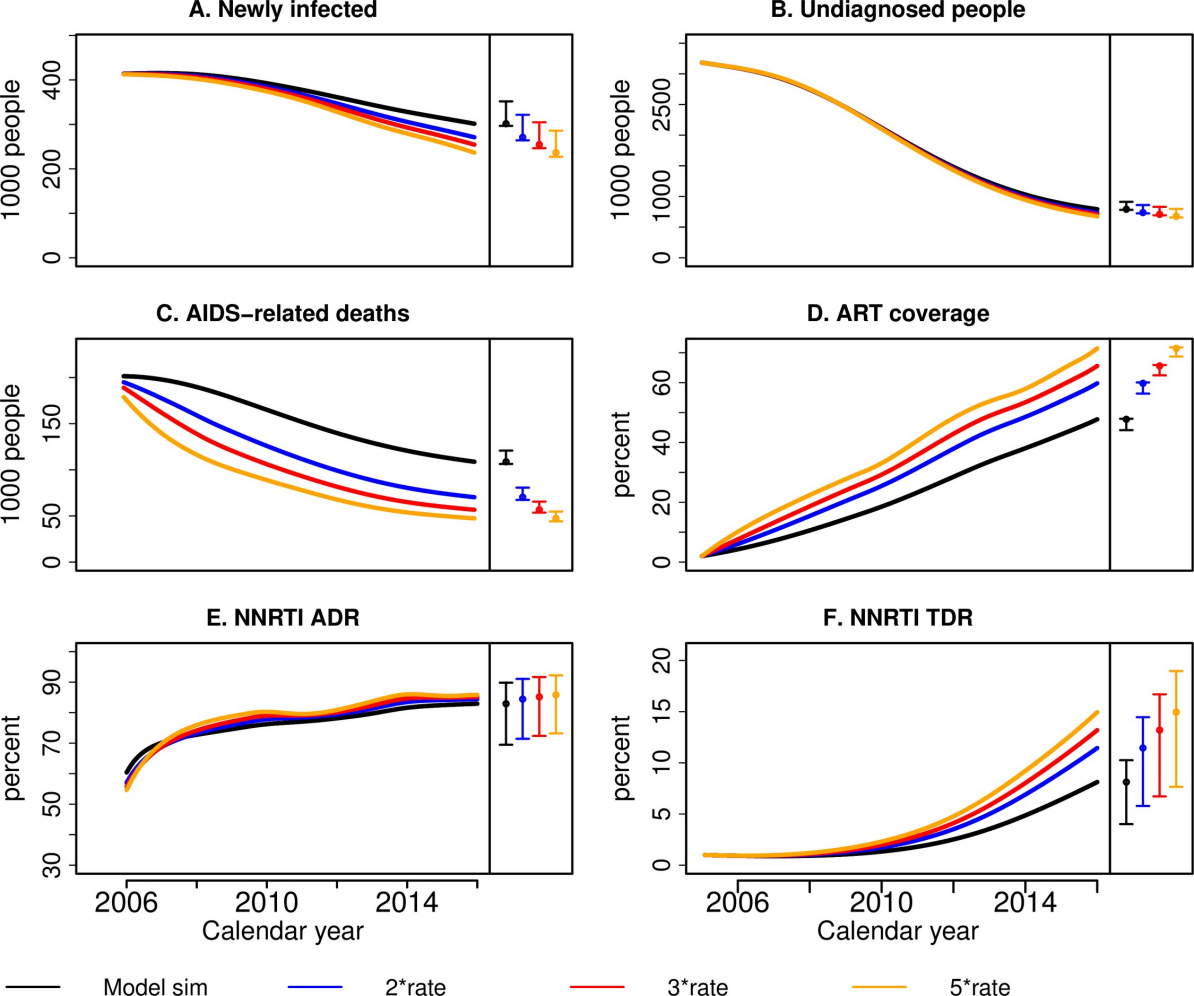
Counterfactual scenario that investigates the impact of increased treatment rate. Simulations of the MARISA model from 2005 to 2016 under the scenarios where the treatment rate is increased by 2, 3 and 5, represented respectively by the blue, red and yellow curves. Simulations of the baseline model are represented in black. The following HIV outcomes are displayed: A) the number of newly infected per year, B) the total number of undiagnosed individuals at each year, C) the number of AIDS-related deaths per year and D) the percentage of infected individuals that are on ART, E) level NNRTI ADR and F) level of NNRTI TDR. Different colours correspond to different rates of starting treatment, where the rates are expressed as multiple of the rate in the standard model. The coloured circles and vertical lines at the right of each sub-figure correspond to the point estimates and 100% sensitivity ranges in 2016, respectively.

In the first scenario, increasing the treatment rate by a factor 2, 3 or 5 during the whole period would have led to a substantial reduction of the number of annual deaths, but would have had little effect on the number of newly-infected or the number of undiagnosed individuals (Fig 3). The decrease in new infections due to increased treatment rates is modest for two reasons: 1) the low proportion of HIV-infected individuals who are ART eligible (only 28% of HIV-infected individuals are diagnosed in 2005) and 2) the decrease in the number of deaths of infectious individuals when increasing ART coverage (67,000 deaths of infectious individuals prevented per year in 2005–2012 under the 5-fold increase scenario). As expected, increasing treatment rates would not have impacted NNRTI ADR levels. On the other hand, by increasing the number of individuals at risk of acquiring NNRTI resistance, it would have led to a considerable increase of NNRTI TDR levels, surpassing 15.0% in 2016 in the 5-fold increase scenario.

**Fig 4:**
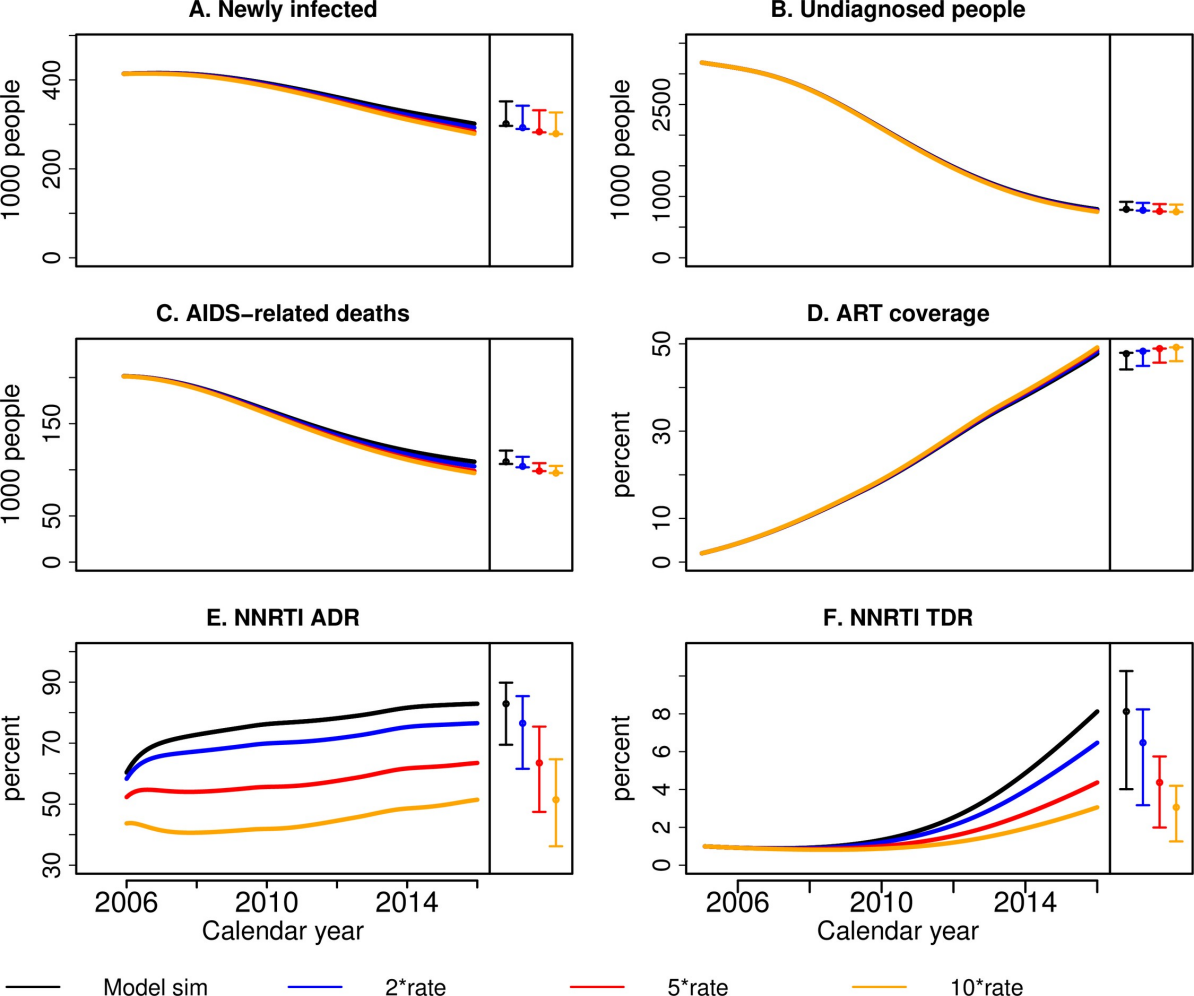
Counterfactual scenario that investigates the impact of increased switching rate to second line regimen. Simulations of the MARISA model from 2005 to 2016 under the scenarios where the switching rate to second-line regimen $\gamma_{F_{1}\to T_{2}}$ is increased by factor 2, 5 and 10, represented respectively by the blue, red and yellow curves. In the baseline simulations represented by the black curves, a switching rate $\gamma_{F_{1}\to T_{2}}$ of 1/2.9 *years*^−1^ is assumed for individuals with CD4<200 copies/μl. The following HIV outcomes are displayed: A) the number of newly infected per year, B) the total number of undiagnosed individuals at each year, C) the number of AIDS-related deaths per year and D) the percentage of infected individuals that are on ART, E) level NNRTI ADR and F) level of NNRTI TDR. Different colours correspond to different rates of starting treatment, where the rates are expressed as multiple of the rate in the standard model. The coloured circles and vertical lines at the right of each sub-figure correspond to the point estimates and 100% sensitivity ranges in 2016, respectively.

In the second scenario, increasing the rate of switching to second-line treatment in case of first-line treatment failure (i.e. dividing the time spent in treatment failure) by factors 2, 5 or 10 would not have influenced the four key HIV outcomes (Fig 4). However, the model predicts a substantial decrease in the levels of both NNRTI ADR and TDR (to 51.5% and 3.1%, respectively), for the 10-fold increase scenario compared to 83% and 8.1%, respectively, for the baseline model in 2016.

Moving the “Treat-All” policy forward in time by 1.5, 3 or 6 years in the third scenario, would have hardly reduced mortality, as it targets individuals with high CD4 counts. On the other hand, removing infectious individuals with high CD4 counts, who are most likely to achieve viral suppression, would have led to a decrease in the number of new infections (256,000 for the 6-year-early implementation scenario instead of 302,000 in the baseline model in 2016). Increasing ART coverage might, however, increase the spread of resistance as NNRTI TDR increased to 10.3% in this scenario.

Finally, in the fourth scenario, drug resistance testing, by directly starting individuals with NNRTI resistance on second-line regimens, would have slightly improved viral suppression among resistant individuals (59.3% instead of 56.9% in the baseline model) and also reduced the transmission of resistance (14.4% instead of 15% resistant among newly infected in 2016). The relative impact of each counterfactual scenario on the number of new infections, AIDS-related deaths and the numbers of both new NNRTI TDR and ADR cases in 2016, as well as their relative percentages is shown in Table 3.

**Table 3:** Impact of counterfactual scenarios on six outcomes: Yearly death, new infections, TDR level, ADR level, number of TDR cases, number of ADR cases, all in 2016. For each of the 6 outcomes and each of the 10 counterfactual scenarios, the absolute difference between the given scenario and baseline model, as well as the 100% sensitivity range (100% SR) are calculated.

Outcome	Scenario 1: Increasing treatment rate			Scenario 2: Increasing switching rate			Scenario 3: Earlier Treat-All			Scenario 4: DR testing
	2∙*γ*	3∙*γ*	5∙*γ*	2∙*γ*	5∙*γ*	10∙*γ*	1.5 years	3 years	5 years	
**Death**										
Mean (per 1000)	-38.5	-52.0	-61.4	-5.0	-10.0	-12.2	-0.0	-1.3	-8.1	-1.1
100% SR	(-41.2,-38.1)	(-56.4,-51.6)	(-67.3,-60.9)	(-6.7,-3.6)	(-13.6,-7.6)	(-16.7,-9.5)	(-0.0,0.0)	(-0.0,0.0)	(-8.5,-7.1)	(-2.9,-0.5)
**New inf.**										
Mean (per 1000)	-30.5	-47.0	-65.0	-8.8	-18.0	-22.4	-5.2	-20.5	-45.8	-2.1
100% SR	(-34.3,-27.0)	(-53.2,-41.9)	(-73.9,-58.6)	(-11.3,-5.5)	(-22.5,-12.1)	(-27.6,-15.7)	(-5.9,-5.1)	(-22.7,-19.8)	(-49.7,-43.4)	(-4.7,-0.9)
**TDR level**										
Mean (in %)	3.3%	5.1%	6.8%	-1.7%	-3.8%	-5.1%	0.1%	0.6%	2.1%	-0.3%
100% SR	(1.8%,4.2%)	(2.7%,6.4%)	(3.6%,8.8%)	(-2.0%,-0.8%)	(-4.5%,-2.0%)	(-6.1%,-2.7%)	(0.1%,0.1%)	(0.4%,0.8%)	(1.1%,2.6%)	(-0.7%,-0.1%)
**ADR level**										
Mean (in %)	1.5%	2.2%	2.9%	-6.4%	-19.4%	-31.5%	-0.7%	0.7%	2.2%	-1.4%
100% SR	(1.0%,2.9%)	(1.4%,4.3%)	(1.8%,5.7%)	(-8.8%,-4.4%)	(-24.3%,-14.3%)	(-36.5%,-25.0%)	(-1.1%,-0.4%)	(0.6%,0.9%)	(1.6%,3.1%)	(-3.9%,-0.8%)
**TDR cases**										
Mean (per 1000)	12.3	17.3	21.2	-9.6	-20.9	-27.7	0.6	3.2	6.0	-2.0
100% SR	(8.6,16.9)	(11.8,24.4)	(14.2,30.9)	(-12.0,-6.2)	(-25.4,-14.4)	(-33.3,-19.3)	(0.3,0.9)	(2.2,4.3)	(4.3,8.3)	(-4.5,-0.8)
**ADR cases**										
Mean (per 1000)	31.8	40.5	47.3	-14.3	-44.7	-74.8	19.4	27.1	7.7	-0.1
100% SR	(29.3,39.0)	(37.7,50.2)	(43.9,58.3)	(-19.0,-10.3)	(-54.0,-34.5)	(-83.1,-62.0)	(14.8,23.0)	(25.1,28.1)	(6.1,11.4)	(-0.3,-0.0)

### Sensitivity analysis

Sensitivity analyses showed that uncertainty in the values of four resistance-related parameters (*σ*_*res*_, *σ*_*rev*_, *α* and the rates of treatment interruption) and of three parameters related to HIV transmission (percentage of MSM, probability of male-to-male infection per sexual contact, and ratio between HIV prevalence in MSM and HET) did not modify substantially the main outcomes of the MARISA model (Fig 2).

## Discussion

In this comprehensive modelling study, we show that the MARISA model captured the dynamics of the HIV epidemic in South Africa over the years 2005–2016. More importantly, it reproduced the emergence of NNRTI resistance, following the roll-out of ART in 2004. The four counterfactual scenarios provided insights into the drivers of NNRTI resistance. They highlighted the close association between the magnitude of ART roll-out and the extent of NNRTI drug resistance. The results also suggest that a better management of first-line treatment failure, improving identification of treatment failure and switching to second-line treatment, might have reduced AIDS-related mortality and new HIV infections, while offering a better control of NNRTI resistance. However, our results also show that while some policies result in substantial reductions in NNRTI TDR, no measure could have stopped its increase. Even with optimal monitoring and management, NNRTI resistance would have rapidly spread in South Africa, suggesting that NNRTI resistance is inevitable if NNRTI-based regimens are used for first-line therapy.

The MARISA model fit was good regarding all four key outcomes of the HIV epidemic in South Africa produced by Thembisa/UNAIDS for the period of study: new infections, number of undiagnosed individuals, AIDS-related deaths and ART coverage [1,17]. The estimates related to the “90-90-90” target provided by our model are also in line with those from UNAIDS. The proportion of HIV-infected individuals knowing their HIV status was estimated at 88% and 86% in 2015 by our model and UNAIDS, respectively. The second “90” was slightly underestimated by the MARISA model: the proportion of individuals with diagnosed HIV infection receiving ART was estimated at 52% in 2015, compared to estimates of 56% and 60% from Thembisa and UNAIDS, respectively. Finally, the proportion of individuals receiving ART achieving viral suppression was estimated at 79%, compared to 78% by UNAIDS [1].

NNRTI ADR and TDR levels estimated by the MARISA model were comparable, though slightly lower, to estimates from six cross-sectional studies conducted during this period [21,27,31–33]. Of note, these observational data were not used for model calibration and the resistance-specific processes of the MARISA model were partly informed using published estimates from other settings (in particular the rate of reversion to a drug-susceptible strain [22] and the positive association between drug resistance and treatment failure [23]), since no data for South Africa were available. Beyond sampling variability in the estimates from the cross-sectional studies, the discrepancy in ADR and TDR estimates between MARISA and the cross-sectional studies could be explained by several factors: a higher proportion of individuals with previous exposure to ART in the studied samples (e.g. through prevention of mother-to-child transmission, not included in the MARISA model), selection bias in the cross-sectional studies (e.g. regarding gender, age, socio-economic features or time since infection), publication bias by which lower measurements of ADR and TDR are less likely to be published, or possibly a misspecification of some parameters of the MARISA model due to geographical differences.

Note that TDR and ADR reflect different populations and processes. ADR is measured in people failing therapy, while TDR is measured in newly diagnosed individuals. The term ADR is somewhat imprecise since we measure it as the proportion of all drug resistant infections among individuals failing treatment and some of these individuals acquired the resistance already by infection. It reflects, however, the terminology used in resource limited settings, where baseline resistance tests are not routinely performed. Our simulations showed that the vast majority of these ADR cases were indeed acquired after treatment failure: in 2016, only 16.9% of ADR cases resulted from treatment failing in individuals already infected with a resistant virus, while the remaining resulted from the selection of resistance mutations in individuals failing on therapy with an initially sensitive virus (see Eq 17 in S1 File). This patterns also explains the relatively weak increase over time (from a high initial level) that is observed for ADR (Fig 2E).

Interestingly, MARISA revealed heterogeneity in NNRTI TDR levels across CD4 strata, with higher levels of NNRTI TDR associated with higher CD4 counts. This can be explained by the fact that individuals with high CD4 counts are more likely to have been recently infected, and thus exposed to a higher risk of NNRTI TDR as the prevalence of NNRTI-resistance increases with time. Other studies have indeed observed a higher NNRTI TDR level among acutely than chronically HIV-infected patients [34]. Given that untreated patients with low CD4 counts might have been infected for a longer time, another explanation could be the increased probability of reversion from a drug-resistant to a wild-type strain in these patients.

The counterfactual scenarios identified two main drivers of the emergence and spread of NNRTI-resistance: the magnitude of the ART roll-out and low frequency of monitoring of first-line treatment failure. The first scenario underlined the inherent risks of resistance emergence induced by a rapid and generalized ART scale-up. This observation is supported by findings of Hamers et al. [35] that the level of NNRTI TDR is associated with time since ART roll out in sub-Saharan Africa. According to the first scenario, policies focused on increasing ART coverage would have allowed a better control of the HIV epidemic, reducing both mortality and new infections. However, such policies would have likely resulted in even higher levels of NNRTI TDR during 2005–2016, leaving doubt about the long-term sustainability of this approach. As seen in the second counterfactual scenario, an earlier treatment switch for individuals failing NNRTI-based treatment would not have prevented resistance from emerging. In this context, the high NNRTI-mutation rate (after on average 6 months in the presence of treatment failure) makes the emergence of NNRTI resistance almost inevitable. For instance, we observe an emergence of TDR (3.3% in 2016) and a substantial level of ADR (50% in 2016), even when assuming that from 2005 an optimal management of treatment failure complying with the South African Department of Health 2016 guidelines [36] was in place. The guidelines recommend a VL measure every six months and an immediate switch to second-line ART after failure of two months of adherence counselling (corresponding to an average time before switching of ${1/\gamma }_{F_{1}\to T_{2}}$ = 5 months). Still, policies focused on improving first-line treatment failure identification and early switching to second-line treatment would have likely led to better control of both the HIV epidemic (with fewer AIDS-related deaths and new infections) and the extent of NNRTI resistance in South Africa. An earlier implementation of the “Treat-All” policy in the third scenario would have modestly decreased mortality, as it extends ART to individuals with high CD4 counts. However, the simulations emphasized the risk of increased levels of NNRTI TDR following implementation of this policy, in a similar way to policies simply increasing ART coverage. Finally, the fourth scenario showed the limited impact on HIV outcomes of implementing drug resistance testing at baseline. Immediate PI-based treatment in patients with TDR only slightly diminished NNRTI TDR prevalence. This small effect may be explained by the limited number of patients affected by the policy (i.e. newly-infected individuals carrying a resistant strain and initiating ART), whose contribution to the transmission of resistance was relatively small (TDR accounts for only 16.9% of ADR cases). We acknowledge that assuming the same failure rates in the counterfactual scenario for patients on a PI-based first-line regimen as for patients on a PI-based second-line regimen may lead to under estimation of the effect of baseline resistance monitoring, because rates of failure in patients on first-line PI-based regimen are probably lower.

The model has several limitations. First, as the estimates from the Thembisa model were used to fit MARISA, findings produced by MARISA partly rely on the accuracy of Thembisa model. Second, it does not take into account NRTI mutations, which could also affect the success of first-line treatment. However, as transmission of NRTI mutations remains at a low level, their impact on the overall effectiveness of first-line regimens is limited [4]. Third, adherence is not modelled explicitly in the model, as it is not systematically assessed in the IeDEA cohorts. Nevertheless, adherence is implicitly included in MARISA, as estimates of suppression and failure rates rely on a large cohort of individuals with different levels of adherence. Moreover, modelling of HIV transmission was based on simplified assumptions: the model only distinguished male from female transmission and attributed two different transmission rates according to awareness of HIV-status. The probability of HIV-infection per sexual act was assumed to be identical for all unsuppressed individuals. The heterogeneity in sexual behavior within genders was only approximated, and MARISA does not account for interactions between resistance status and sexual behavior. However, in view of the good fit to the number of new infections, there is no need for introducing a more complex representation of HIV transmission dynamics. Finally, the model does not simulate prevention of mother-to-child transmission, which could be an important source of NNRTI resistance. Overall, there is a trade-off between these potential additional layers of complexity and the limited knowledge about specific mechanisms. We argue that the ability of the MARISA model to capture the dynamics of NNRTI resistance with parameters fixed to known values from external data supports the validity of these simplifications. As it stands, the model does not make any unverifiable assumptions, and the sensitivity analyses showed that conclusions were robust, despite uncertainty in the main parameters related to resistance and transmission. Furthermore, the relatively simple representation of NNRTI resistance emergence and transmission makes the model easily interpretable.

MARISA can be adapted to address other questions on HIV drug resistance by adding further layers of complexity. The imminent roll-out of Dolutegravir (DTG) has been presented as a response to the NNRTI resistance epidemic [5]. In South Africa, DTG in combination with two NRTI-class drugs will progressively replace NNRTI as the first-line regimen for men, but there is uncertainty as to whether it should be recommended for women of reproductive age due to safety issues [37]. DTG will also be prescribed to patients failing NNRTI-based regimens. As NRTI resistance mutations might already have occurred in these patients, this could affect the future success of the DTG-based regimen [38,39]. From this basis, MARISA can be extended in order to evaluate the potential impact of introducing DTG-based regimens, either for men and women or for men only. While the overall structure of the model in terms of care and disease progression will stay unchanged, the resistance dimension can be expanded by adding key NRTI-resistance mutations (e.g. K65R and M184V). We could also stratify the model by age group in order to represent the difference in drug prescription (NNRTI or DTG) in women according to age. MARISA could thus be used to predict the spread of NRTI- and NNRTI-resistance mutations according to the different strategies of DTG roll-out and their impact on the overall success of HIV-epidemic.

To conclude, we propose MARISA, a mechanistic model aimed at providing insight into the NNRTI resistance epidemic in South Africa in 2005–2016. Integrating information from several sources, including local cohorts of HIV-infected individuals, the model captured the essence of NNRTI resistance emergence in South Africa. Counter-factual scenarios identified key drivers of the NNRTI resistance epidemic at the policy level: a rapid, large-scale ART roll-out and an insufficient monitoring of first-line treatment failure. The model also showed that the rapid rate of acquisition and slow rate of reversion of NNRTI drug resistance mutations make it difficult to prevent their spread if NNRTI-based treatments are used as a first-line regimen, and it indicated the limited effect of drug resistance testing. Understanding future challenges in HIV drug resistance such as the introduction of DTG, its effect on the epidemic, the possibility of DTG resistance, and the impact of NRTI mutations on DTG based regimens will require the modelling of a more complex and uncertain mutational landscape. MARISA, with its backbone of a simple yet adequate epidemiological model will provide a suitable foundation to address this challenge.

## Supporting information

S1 File Detailed description of the MARISA model. Detailed description of the MARISA model and the computational methods used to calibrate and then run it.
(PDF)
