# Supplementary material for: Bridging the gap between HIV epidemiology and antiretroviral resistance evolution: Modelling the spread of resistance in South Africa
Source: PLoS Comput Biol. 2019 Jun 24;15(6):e1007083. doi: 10.1371/journal.pcbi.1007083 (PMC6611642; doi:10.1371/journal.pcbi.1007083)
Supplement: S1 File — Detailed description of the MARISA model and the computational methods used to calibrate and then run it. (PDF) [file pcbi.1007083.s001.pdf]

Supplementary material

Bridging the gap between HIV epidemiology and antiretroviral resistance evolution: Modelling the spread of resistance in South Africa

Anthony Hauser & al.

June 8, 2019

Contents

1 Model structure **1**
1.1 Modelling HIV transmission 2
1.2 Modelling mortality 3
1.3 Modelling diagnosis and treatment rate 3
1.4 Modelling resistance 4
1.5 Modelling demographic changes 5

2 Model equations **6**
2.1 Notations 6
2.2 ODEs 9
2.3 Starting values 10

3 Calibration and model simulation **10**
3.1 Survival analysis 10
3.2 Likelihood maximisation 12

4 Results **14**
4.1 Best fits 14
4.2 Sensitivity analysis 14

1 Model structure

The model is split in 4 dimensions: 1) care stages (8 levels), 2) disease progression, characterised by the CD4 counts (4 levels), 3) NNRTI resistance, and 4) gender. The eight care stages are "infected but not diagnosed" (I), "diagnosed but not treated" (D), "started treatment" (T_1 and

T_2, respectively for first and second-line treatment), "suppressed" (S_1 and S_2) and "failed" (F_1 and F_2). An individual is considered as having "started treatment" (T_1 or T_2) if he initiated treatment less than 3 months ago. Afterwards, he/she is considered either as suppressed if VL < 1000 cp/ml, or as failed otherwise. The four different CD4 strata are represented by the letter i in the equations and are respectively: $CD4 > 500 cells/\mu l$ ($i = 1$), $350 < CD4 < 500c/\mu l$ ($i = 2$), $200 < CD4 < 350c/\mu l$ ($i = 3$) and $CD4 < 200c/\mu l$ ($i = 4$). NNRTI resistance is represented by j, and its value is 0 if an individual is susceptible to NNRTI and 1 otherwise. The gender dimension, represented by k, takes 0 for male and 1 for female. The indices i, j and k are used in equations in order to specify a particular layer of each dimension. When an index is missing, it means that we have summed over all the layers that the dimension contains (e.g. $I^k(t) := \sum_{i,j} I^{i,j,k}(t)$).

1.1 Modelling HIV transmission

The number of newly infected individuals per time step (1 month) is split in three parts, characterising the three different transmission routes: from men to men (men who have sex with men - MSM), from men to women and from women to men (heterosexual transmission - HET). Let k and k' respectively be the gender of a susceptible (HIV-negative) and an infected individual. Assuming a density-dependent transmission and different risk behaviours between infected individuals knowing or not their status, the number of individuals of gender k that have been newly infected by individuals of gender k' is:

$$\Delta_{k,k'} = \beta_u \nu_{k,k'} \frac{I^{k',k}}{N^{k',k}} Susc^{k,k'} + \beta_d \nu_{k,k'} \frac{D^{k',k} + T_1^{k',k} + F_1^{k',k} + T_2^{k',k} + F_2^{k',k}}{N^{k',k}} Susc^{k,k'}, \quad (1)$$

where $I^{k,k'}$, $Susc^{k,k'}$ and $N^{k,k'}$ represent respectively the number of infected people, the number of susceptible people and the total number of people of gender k that have unprotected intercourse with the gender k', β_d and β_u the frequency of unprotected intercourse per month, respectively for infected people knowing their status (diagnosed) and for those who do not (undiagnosed) and $\nu_{k,k'}$ the probability that an unprotected intercourse leads to a new infection between an infected individual of gender k' and a susceptible individual of gender k.
As HET and MSM populations were not formally split in the model, we approximated $\frac{I^{k',k}}{N^{k',k}}$ by $\frac{I^{k'}}{N^{k'}}$ (where $I^{k'} := \sum_k I^{k',k}$ and $N^{k'} := \sum_k N^{k',k}$), assuming a similar HIV prevalence in MSM as in the overall male population. We also replaced $Susc^{k,k'}$ by $\rho_{k,k'} Susc^k$, where $\rho_{k,k'}$ represents the proportion of people having sexual intercourse with gender k' among sexually active people of gender k and where $Susc^k = Susc^{k,0} + Susc^{k,1}$. We assumed that $\nu_{1,1} = 0$ as there is no risk of infection during a sexual intercourse between two women. The proportion of MSM among men $\rho_{0,0}$ is 0.05, as reported by Anova Health Foundation [1]. Similarly, we set the proportion of sexually active women that have a sexual intercouse with men $\rho_{1,0}$ to 0.95. Using Eq 1, the number of newly infected individuals of gender k per time step is:

$$\begin{aligned} \Delta_k =& \Delta_{k,0} + \Delta_{k,1} \\ =& \beta_u \nu_{k,0} \rho_{k,0} \frac{I^{k'=0}}{N^{k'=0}} Susc^k + \beta_d \nu_{k,0} \rho_{k,0} \frac{D^{k'=0} + T_1^{k'=0} + F_1^{k'=0} + T_2^{k'=0} + F_2^{k'=0}}{N^{k'=0}} Susc^k \\ &+ \beta_u \nu_{k,1} \rho_{k,1} \frac{I^{k'=1}}{N^{k'=1}} Susc^k + \beta_d \nu_{k,1} \rho_{k,1} \frac{D^{k'=1} + T_1^{k'=1} + F_1^{k'=1} + T_2^{k'=1} + F_2^{k'=1}}{N^{k'=1}} Susc^k. \end{aligned} \quad (2)$$

We assumed that all newly infected individuals arrive at the first CD4 stratum ($i = 1$). See Eq 18 for more details.

1.2 Modelling mortality

Mortality rates differ according to CD4 counts and care stages. We combined the results of two studies in order to estimate the relative risks for each group[2, 3]. The group of suppressed individuals with $CD4 > 500c/\mu l$ is defined as the reference as they have the lowest risk. To convert the relative risks into rates, we used a free parameter μ, which corresponds to the mortality rate of the reference group (see Table 5).
As [2] observed a high heterogeneity in mortality risk among people with $CD4 < 200c/\mu l$ depending on CD4 counts, mortality was modelled differently for this class, based on what has already been done in Thembisa model [4]. Instead of assuming a fixed risk as for the other classes, we allowed the mortality rate of the last CD4 stratum ($CD4 < 200c/\mu l$) to vary within a range, according to its proportion of people with very low CD4 counts. The upper level of the range corresponds to the relative risk in the scenario where all individuals have $CD4 < 50c/\mu l$, while the lower level corresponds to the scenario where all individuals have $CD4 > 50c/\mu l$. As we do not have any accurate information over time about this proportion, we used rate of ART initiation as a proxy. We assumed that average treatment rate of the 3 previous years determines the proportion of individuals with $CD4 > 50c/\mu l$, as shown in Eq 3. This average corresponds to the increase in treatment rate relative to 2005. We assumed an exponential decrease of the proportion of people with $CD4 < 50c/\mu l$ when the treatment rate is increasing, in line which structure of the compartmental model assuming exponential distribution of the rates.

$$\begin{aligned} p_{CD4>50}(t) &= p_{CD4>50}(2005) \cdot \exp\left(q \cdot \text{"diff. in 3-year Average"}(\gamma^4_{D\to T_1}(t))\right) \\ &= p_{CD4>50}(2005) \cdot \exp\left(q \cdot \left(\sum_{s=1}^{36} \gamma^4_{D\to T_1}\left(t - \frac{s}{12}\right) - \sum_{s=1}^{36} \gamma^4_{D\to T_1}\left(2005 - \frac{s}{12}\right)\right)\right), \end{aligned} \tag{3}$$

where $p_{CD4>50}(0) := 27\%$ is the proportion of people with $CD4 > 50c/\mu l$ [5]. Therefore, the relative mortality risk for people with $CD4 < 200c/\mu l$ is

$$\mu_{CD4<200}(t) = p_{CD4>50}(t) \cdot \mu_{50<CD4<200}(t) + (1 - p_{CD4>50}(t)) \cdot \mu_{CD4<50}(t),$$

where $\mu_{50<CD4<200}(t)$ and $\mu_{CD4<50}(t)$ are relative mortality risks and represent respectively the lower and upper bounds of $\mu_{CD4<200}(t)$ (see Table 4).

1.3 Modelling diagnosis and treatment rate

Diagnosis rate
To model the diagnosis rate, we needed to distinguish three different types of testing: 1) testing asymptomatic individuals γ^k_{diag1}, 2) testing symptomatic (opportunistic infection - OI) γ^i_{diag2} and 3) testing pregnant women $\gamma^{i,k}_{diag3}$. The overall diagnosis rate for a given CD4 stratum i and gender k is thus: $\gamma^{i,k}_{I\to D} = \gamma^k_{diag1} + \gamma^i_{diag2} + \gamma^{i,k}_{diag3}$ [6].
The diagnosis rate γ^k_{diag1} has increased over the years, as a consequence of the augmentation in the number of HIV-tests performed (in the asymptomatic population). To model the increase over time, two free parameters are included into the diagnosis rate: one representing the diagnosis rate in 2005 for men, and one representing its increase between 2005 and 2015. We assumed similar diagnosis rates for the four CD4 strata. As reported in [6], diagnosis rate varies across gender, being higher for women. The diagnosis for women is thus: $\gamma^{k=1}_{diag1} = \gamma^{k=0}_{diag1}/p_{I\to D}$, where $p_{I\to D}$ is a fixed parameter [6].
The rate of diagnosis due to OI γ^i_{diag2} depends on CD4 counts as OI is more likely to occur with low CD4 counts. Following what has been done by Thembisa [4], we set γ^i_{diag2} as: $\gamma^i_{diag2} = inc^i_{OI} \cdot \gamma_{test2}(t)$, where inc^i_{OI} represents the OI incidence and is set as $0.05/(1000 person \cdot year)$ for

ind. with $CD4 > 500c/\mu l$, $0.12/(1000py)$ for ind. with $350 < CD4 < 500c/\mu l$, $0.27/(1000py)$ for ind. with $200 < CD4 < 350c/\mu l$, $0.9/(1000py)$ for ind. with $CD4 < 200c/\mu l$. $\gamma_{test2}(t)$ represents the monthly testing rate for individuals having an OI. To model its increase over time, we used a sigmoid function increasing from 2% in 2005 to 8% in 2015 [4].
$\gamma^{i,k}_{diag3}$ model the increased diagnosis rate due to pregnancy. It is set as 0 for men and decreases with CD4 counts, as fertility rate is lower for women with low CD4 counts. This rate is: $\gamma^{i,k}_{diag3}(t) = \theta_{birth} \cdot \theta^{i}_{birth\ CD4} \cdot \gamma^{k}_{test3}(t)$, where $\theta_{birth} = 23/(1000py)$ is the birth rate in the overall South African population, $\theta^{i}_{birth\ CD4}$ the decreased in birth rate according to CD4 counts and $\gamma^{k}_{test3}(t)$ the monthly testing rate for pregnant women [4]. $\theta^{i}_{birth\ CD4}$ is 1 for women with $CD4 > 500c/\mu l$, 0.96 for women with $350 < CD4 < 500c/\mu l$, 0.87 for women with $200 < CD4 < 350c/\mu l$, 0.74 for women with $CD4 < 200c/\mu l$. We used a sigmoid function to model to model the monthly testing rate for pregnant women. This increases from 4% in 2005 to 8% in 2010 [4].

Treatment rate
We allowed treatment rate to vary over time and CD4 classes. The treatment rate for the CD4 class i at time t is :

$$\gamma^{i}_{D\to T_1}(t) = \gamma^{CD4<200}_{2005} \cdot r^{elig}_{i}(t) \cdot r^{CD4}_{i} \cdot r^{time}(t), \tag{4}$$

where $\gamma^{CD4<200}_{2005}$ is a free parameter representing the treatment rate for an eligible individual in 2005 with $CD4 < 200c/\mu l$, $r^{elig}_{i}(t)$ the proportion of individuals assumed to be eligible within CD4 class i at time t, $r^{CD4}_{i}(t)$ the relative treatment rate for CD4 class i relative to that in $CD4 < 200c/\mu l$ class ($i = 4$), $r^{time}(t)$ the relative treatment rate at time t relative to that in 2005.
Aside from the free parameter $\gamma^{CD4<200}_{2005}$, the values of all other parameters from Eq 4 are taken from the Thembisa model [4]. We set r^{CD4}_{i} to 0.4 for the $CD4 > 500c/\mu l$ class ($i = 1$), 0.5 for the $500 > CD4 > 350c/\mu l$ class ($i = 2$), 0.7 for the $350 > CD4 > 200c/\mu l$ class ($i = 3$) and 1 (reference group) for the $CD4 < 200c/\mu l$ class ($i = 4$). The parameter $r^{elig}_{i}(t)$ models the broadening of eligibility criteria over time as well as the delay between guideline change and change in practice. The product $r^{elig}_{i}(t) \cdot r^{CD4}_{i}(t)$ is displayed in Figure 1. We used a sigmoid function in order to model the gradual increase of the treatment rate over time, represented by $r^{time}(t)$ in the equation. Based on Thembisa data [4, p.28], the function $r^{time}(t)$ implies a 17-fold increase between 2002 and 2012. Initiation of PI-based treatment as a first-line regimen is represented by the rates $\gamma^{i}_{D\to T_2}$. These rates has been estimated using IeDEA-SA data (see section 3.1).

1.4 Modelling resistance

The resistance dimension consists of two layers: "being NNRTI-susceptible" and "being NNRTI-resistant". Individuals can enter the resistant layer in two different ways: either by acquiring drug resistance or by being infected by a resistant strain. Acquisition of NNRTI resistance at a rate σ_{res} is only possible when failing the first-line treatment. Alternatively, there is a risk to be directly infected by a resistant virus, this risk being proportional to the percentage of infectious individuals that are resistant. Resistant individuals can also revert back to the susceptible layer at a rate σ_{rev} when no more drug pressure is exerted. As time to virological failure is lower for resistant individuals [7], we added a fixed parameter α in order to express this difference. It represents the hazard ratio of the time to virological suppression (susceptible vs resistant) and has been collected from literature [7]. Similar differences have been observed in time to viral failure between susceptible and resistant individuals. For the sake of simplicity, we decided to use the same parameter α to model both differences, as estimates found in the literature were

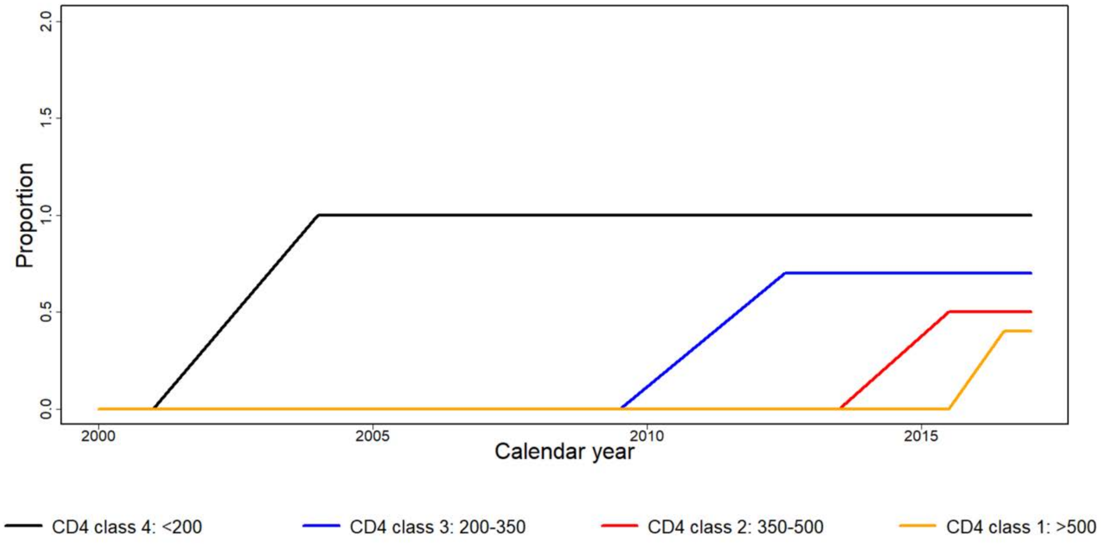

Figure 1: Proportion of eligible individuals across CD4 classes over time multiplied by relative treatment rates (relative to the $CD4 < 200$ class): $r_i^{elig}(t) \cdot r_i^{CD4}$.

within the same range.

1.5 Modelling demographic changes

Susceptible people

As the model only describes the HIV-infected population, the susceptible population $Susc^k(t)$ is not part of the model. However, as $Susc_k(t)$ is needed in order to model infections, it is estimated as follows: $Susc^k(t) = N^k(t) - Inf_k(t)$, where $N^k(t)$ is the total number of adults of gender k in South Africa estimated by the World Health Organisation (WHO) [8] and $Inf^k(t)$ is the total number of infected adults of gender k calculated by the model (sum over all compartments). We used demographic data and interpolated them in order to obtain a function $N^k(t)$ that is continuous over time.

Children reaching adulthood

The inflow of children reaching adulthood ($\geq$ 15 years old) was calculated using [4], [9] and [10]. Thembisa model provides yearly estimates of the number of 15-year olds that are 1) HIV-infected, 2) diagnosed and 3) on ART, stratified by gender. NNRTI resistance prevalence in 15-year olds on ART were estimated by only considering acquisition of NNRTI-resistance during ART. Transmission of resistance due to the prevention of mother to child transmission (PMTCT) treatment was not considered, as the national PMTCT programme only started in 2002 in South Africa, and thus does not have any effect on 15-year olds before 2017. Moreover, we did not consider transmission of resistance from mother to child, as level of resistance was very low in 2002. We considered that 20% of 15-year olds were on a failing ART [9]. Among them, we considered that 90% were resistant to NNRTI [10]. We interpolated these yearly estimates into continuous monthly estimates.

2 Model equations

2.1 Notations

Eq 6-9 model the difference in treatment failure/success rates depending on the absence or presence of NNRTI-resistance. Table 1 shows the different compartments of the model and model outcomes (see also Eq 10-17).

$$\delta(j) = \begin{cases} -1 & \text{if } j = 0 \\ 1 & \text{if } j = 1 \end{cases} \tag{5}$$

Impact of resistance on clinical outcome

$$\gamma^{i,j=1}_{T_1 \to S_1}(t) = 1/\alpha \cdot \gamma^{i,j=0}_{T_1 \to S_1}(t) \tag{6}$$

$$\gamma^{i,j=1}_{T_1 \to F_1}(t) = \alpha \cdot \gamma^{i,j=0}_{T_1 \to F_1}(t) \tag{7}$$

$$\gamma^{i,j=1}_{F_1 \to S_1}(t) = 1/\alpha \cdot \gamma^{i,j=0}_{F_1 \to S_1}(t) \tag{8}$$

$$\gamma^{i,j=1}_{S_1 \to F_1}(t) = \alpha \cdot \gamma^{i,j=0}_{S_1 \to F_1}(t) \tag{9}$$

Number of newly infections between time $s-1$ and s (see Eq.2)

$$\begin{aligned} \Delta Inf(s) = \int_{s-1}^{s} \Bigg(& \beta_u \sum_{k=0}^{1} \left(\rho_{k,1-k}\nu_{k,1-k} \frac{Inf_u^{1-k}}{N^k} Susc^k + \rho_{k,k}\nu_{k,k} \frac{Inf_u^{k}}{N^k} Susc^k \right)(t) \\ & + \beta_d \sum_{k=0}^{1} \left(\rho_{k,1-k}\nu_{k,1-k} \frac{Inf_d^{1-k}}{N^k} Susc^k + \rho_{k,k}\nu_{k,k} \frac{Inf_d^{k}}{N^k} Susc^k \right)(t) \Bigg) dt \end{aligned} \tag{10}$$

Number of diagnosed HIV-infected individuals at time t

$$Diag(t) = D(t) + T_1(t) + S_1(t) + F_1(t) + T_2(t) + S_2(t) + F_2(t) \tag{11}$$

Number of AIDS-related deaths between time $s-1$ and s

$$Mort(s) = \int_{s-1}^{s} \left(\mu_I^{i=4} \cdot I^{i=4} + \mu_D^{i=4} \cdot D^{i=4} + \mu_{T_1}^{i=4} \cdot T_1^{i=4} + \ldots + \mu_{F_2}^{i=4} \cdot F_2^{i=4} \right)(t) dt \tag{12}$$

Number of individuals on ART at time t

$$Treat(t) = T_1(t) + S_1(t) + \ldots + F_2(t) \tag{13}$$

Number of infectious and diagnosed individuals

$$Inf_d^{jk}(t) = D^{jk}(t) + T_1^{jk}(t) + F_1^{jk}(t) + T_2^{jk}(t) + F_2^{jk}(t) \tag{14}$$

Notation	Description	Definition
Dimensions/Compartments		
i	index for the 2nd dimension (CD4 counts)	$i = 1, 2, 3, 4$ (4 CD4 strata)
j	index for the 3rd dimension (resistance)	$j = 0$: NNRTI-susceptible
		$j = 1$: NNRTI-resistant
k	index for the 4th dimension (gender)	$k = 0$: men, $k = 1$: women
$I^{ijk}(t)$	number of infected (not diagnosed) indiv.	
$D^{ijk}(t)$	number of diagnosed (not treated) indiv.	
$T_1^{ijk}(t)$	number of indiv. that have started 1st	
	line treatment for less than 3 months	
$S_1^{ijk}(t)$	number of suppressed indiv.	
	on 1st-line treatment	
$F_1^{ijk}(t)$	number of indiv. failing 1st-line treatment	
$T_2^{ijk}(t)$	number of indiv. that have started 2nd	
	line treatment for less than 3 months	
$S_2^{ijk}(t)$	number of suppressed indiv.	
	on 2nd-line treatment	
$F_2^{ijk}(t)$	number of indiv. failing 2nd-line treatment	
$N^k(t)$	number of adults of gender k	[8]
$Susc^k$	number of susceptible indiv. of gender k	by definition:
		$Susc^k := N^k(t) - I^k(t)$.
$Inf_u^{jk}(t)$	number of undiagnosed indiv.	$Inf_u^{jk}(t) := I^{jk}(t)$
$Inf_d^{jk}(t)$	number of infectious diagnosed indiv.	see Eq 14
Model outcomes		
$\Delta Inf(t)$	number of newly infected indiv. between times t	see equation 10
	and $t + \Delta t$ ($\Delta(t)$ is the time step: 1 month)	
$Diag(t)$	number of indiv. diagnosed	see Eq 11
$Mort(t)$	number of AIDS-related deaths	see Eq 12
$Treat(t)$	number of indiv. treated	see Eq 13
$ADR(t)$	level of ADR (among failed indiv.)	see Eq 15
$TDR(t)$	level of TDR (among newly diagnosed indiv.)	see Eq 16

Table 1: Description of the compartments and dimensions of the model.

Acquired NNRTI resistance (in failing patients)

$$ADR(t) = \frac{F_1^{j=1}(t)}{\sum_{j=0}^{1} F_1^{j}(t)} \tag{15}$$

Transmitted NNRTI (in newly diagnosed individuals)

$$TDR(t) = \frac{\Delta D^{j=1}(t)}{\sum_{j=0}^{1} \Delta D^{j}(t)} \tag{16}$$

where $\Delta D^j(t)$ represent the inflow of individuals going from I^j to D^j at each time step (newly diagnosed individuals).

Contribution of TDR to ADR between times $s-1$ and s (see Discussion section in the manuscript)

$$\rho_{TDR \to ADR}(s) = \frac{\sum_{i=1}^{4} \int_{s-1}^{s} \left(\gamma_{S_1 \to F_1}^{i,j=1} \cdot S_1^{i,j=1}(t) + \gamma_{T_1 \to F_1}^{i,j=1} \cdot T_1^{i,j=1}(t) \right) dt}{\sum_{i=1}^{4} \int_{s-1}^{s} \left(\gamma_{S_1 \to F_1}^{i,j=1} \cdot S_1^{i,j=1}(t) + \gamma_{T_1 \to F_1}^{i,j=1} \cdot T_1^{i,j=1}(t) + \sigma_{res} \cdot F_1^{i,j=0}(t) \right) dt} \tag{17}$$

2.2 ODEs

$$
\begin{aligned}
\dot{I}^{ijk}(t) =& -\nu_{CD4}^{I,i}\cdot I^{ijk}(t)\mathbb{1}_{i\le 3}+\nu_{CD4}^{I,i-1}\cdot I^{(i-1)jk}(t)\mathbb{1}_{i\ge 2}\\
&+\beta_u\left(\rho_{k,1-k}\nu_{k,1-k}\frac{Inf_u^{ij(1-k)}}{N^k}Susc^k+\rho_{k,k}\nu_{k,k}\frac{Inf_u^{ijk}}{N^k}Susc^k\right)\mathbb{1}_{i=1}\\
&+\beta_d\left(\rho_{k,1-k}\nu_{k,1-k}\frac{Inf_d^{ij(1-k)}}{N^k}Susc^k+\rho_{k,k}\nu_{k,k}\frac{Inf_d^{ijk}}{N^k}Susc^k\right)\mathbb{1}_{i=1}\\
&-\gamma_{I\to D}^{ik}(t)\cdot I^{ijk}(t)-\delta(j)\cdot\sigma_{rev}\cdot I^{i1k}(t)-\mu_I^i\cdot I^{ijk}(t)+\Delta Inf_{15years}^{I,ijk}(t),\\
\dot{D}^{ijk}(t) =& -\nu_{CD4}^{D,i}\cdot D^{ijk}(t)\mathbb{1}_{i\le 3}+\nu_{CD4}^{D,1}\cdot D^{(i-1)jk}(t)\mathbb{1}_{i\ge 2}-(\gamma_{D\to T_1}^{ik}(t)+\gamma_{D\to T_2}^{i}(t))\cdot D^{ijk}(t)\\
&+\gamma_{T_1\to D}^{i}\cdot T_1^{ijk}(t)+\gamma_{S_1\to D}^{i}\cdot S_1^{ijk}(t)+\gamma_{F_1\to D}^{i}\cdot F_1^{ijk}(t)+\gamma_{I\to D}^{ik}(t)\cdot I^{ijk}(t)\\
&-\delta(j)\cdot\sigma_{rev}\cdot D^{i1k}(t)-\mu_D^i\cdot D^{ijk}(t)+\Delta Inf_{15years}^{D,ijk}(t),\\
\dot{T}_1^{ijk}(t) =& \left(\nu_{CD4}^{T_1,i-1}\cdot T_1^{(i-1)jk}(t)-\tilde{\nu}_{CD4}^{T_1,i-1}\cdot T_1^{ijk}(t)\right)\mathbb{1}_{i\ge 2}+\left(\tilde{\nu}_{CD4}^{T_1,i}\cdot T_1^{(i+1)jk}(t)-\nu_{CD4}^{T_1,i}\cdot T_1^{ijk}(t)\right)\mathbb{1}_{i\le 3}\\
&-(\gamma_{T_1\to S_1}^{ij}+\gamma_{T_1\to F_1}^{ij}+\gamma_{T_1\to D}^{ij})\cdot T_1^{ijk}(t)+\gamma_{D\to T_1}^{ik}(t)\cdot D^{ijk}(t)-\mu_{T_1}^i\cdot T_1^{ijk}(t)+\Delta Inf_{15years}^{T_1,ijk}(t),\\
\dot{S}_1^{ijk}(t) =& -\tilde{\nu}_{CD4}^{S_1,i-1}\cdot S_1^{ijk}(t)\mathbb{1}_{i\ge 2}+\tilde{\nu}_{CD4}^{S_1,i}\cdot S_1^{(i+1)jk}(t)\mathbb{1}_{i\le 3}\\
&-(\gamma_{S_1\to F_1}^{ij}+\gamma_{S_1\to D}^{ij})\cdot S_1^{ijk}(t)+\gamma_{T_1\to S_1}^{ij}\cdot T_1^{ijk}(t)+\gamma_{F_1\to S_1}^{ij}\cdot S_1^{ijk}(t)-\mu_{S_1}^i\cdot S_1^{ijk}(t),\\
\dot{F}_1^{ijk}(t) =& \nu_{CD4}^{F_1,i-1}\cdot F_1^{(i-1)jk}(t)\mathbb{1}_{i\ge 2}-\nu_{CD4}^{F_1,i}\cdot F_1^{ijk}(t)\mathbb{1}_{i\le 3}+\delta(j)\cdot\sigma_{res}\cdot F_1^{i0k}(t)\\
&-(\gamma_{F_1\to S_1}^{ij}+\gamma_{F_1\to T_2}^{ik}+\gamma_{F_1\to D}^{ij}(t))\cdot F_1^{ijk}(t)+\gamma_{S_1\to F_1}^{ij}\cdot S_1^{ijk}(t)+\gamma_{T_1\to F_1}^{ij}\cdot T_1^{ijk}(t)\\
&-\mu_{F_1}^i\cdot F_1^{ijk}(t),\\
\dot{T}_2^{ijk}(t) =& \left(\nu_{CD4}^{T_2,i-1}\cdot T_2^{(i-1)jk}(t)-\tilde{\nu}_{CD4}^{T_2,i-1}\cdot T_2^{ijk}(t)\right)\mathbb{1}_{i\ge 2}+\left(\tilde{\nu}_{CD4}^{T_2,i}\cdot T_2^{(i+1)jk}(t)-\nu_{CD4}^{T_2,i}\cdot T_2^{ijk}(t)\right)\mathbb{1}_{i\le 3}\\
&-(\gamma_{T_2\to S_2}^{ij}+\gamma_{T_2\to F_2}^{ij})\cdot T_2^{ijk}(t)+\gamma_{F_1\to T_2}^{ik}\cdot F_1^{ijk}(t)+\gamma_{D\to T_2}^{i}\cdot D^{ijk}(t)-\mu_{T_2}^i\cdot T_2^{ijk}(t),\\
\dot{S}_2^{ijk}(t) =& -\tilde{\nu}_{CD4}^{S_2,i-1}\cdot S_2^{ijk}(t)\mathbb{1}_{i\ge 2}+\tilde{\nu}_{CD4}^{S_2,i}\cdot S_2^{(i+1)jk}(t)\mathbb{1}_{i\le 3}\\
&-\gamma_{S_2\to F_2}^{ij}\cdot S_2^{ijk}(t)+\gamma_{T_2\to S_2}^{ij}\cdot T_2^{ijk}(t)+\gamma_{F_2\to S_2}^{ij}\cdot F_2^{ijk}(t)-\mu_{S_2}^i\cdot S_2^{ijk}(t),\\
\dot{F}_2^{ijk}(t) =& \nu_{CD4}^{F_2,i-1}\cdot F_2^{(i-1)jk}(t)\mathbb{1}_{i\ge 2}-\nu_{CD4}^{F_2,i}\cdot F_2^{ijk}(t)\mathbb{1}_{i\le 3}\\
&-\gamma_{F_2\to S_2}^{ij}\cdot F_2^{ijk}(t)+\gamma_{S_2\to F_2}^{ij}\cdot S_2^{ijk}(t)+\gamma_{T_2\to F_2}^{ij}\cdot T_2^{ijk}(t)-\mu_{F_2}^i\cdot F_2^{ijk}(t).
\end{aligned}
\tag{18}
$$

2.3 Starting values

Model simulation started in 2005. The distribution of the infected individuals over the 128 compartments at the start of the simulation were chosen as follows. As reported by Thembisa, there were 4.4 million adult people living with HIV in 2005 in South Africa, 1.7 million of them were men. These individuals were distributed across 3 of the 8 care stages: infected (I), diagnosed (D) and treated with first-line (T_1). 72% were in I, 26% in D and 2% in T_1. The distribution of individuals across the four CD4 classes has been determined by running with different distributions across CD4 classes and by keeping the one with the best fit. For infected I individuals, we chose a homogeneous distribution, i.e. the same number of individuals in the four CD4 classes. For diagnosed and treated individuals (D and T_1), the fourth CD4 class ($CD4 < 200c/\mu l$) has the most individuals. It has twice as many individuals as in the third CD4 class, which has twice as many as in the second CD4 class, which has twice as many as in the first one. Finally, we assumed that 1% of infected individuals were resistant to NNRTI. For sake of simplicity, this percentage did not depend on CD4 counts or on care stages.

3 Calibration and model simulation

We calibrated the model in two successive steps. First, IeDEA data were used to estimate the majority of the rates with survival analysis. Second, we fitted our model to Thembisa data using a maximum likelihood approach. A few parameters, mainly the ones related to resistance, were collected from literature. For more details, see Table 4.

3.1 Survival analysis

To estimate the majority of the rates that are related to continuum of care or disease progression, we used data from IeDEA-SA, a network collecting individual information about HIV-infected patients from several cohorts in Southern Africa. After having selected only adults from the 5 South African cohorts and discarded patients with erroneous information, we ended up with information about 54'016 patients. This includes 1) start/end of drug regimen, 2) VL measurements, 3) CD4 counts measurements and 4) outcome (i.e. death).
Let A and B be two compartments of the model and $r_{A\to B}$ the rate corresponding to the movement from A to B. When a linear interaction ($\frac{dB}{dt} = r_{A\to B} \cdot A$) is chosen, the compartmental model assumes that the *time $T_{A\to B}$ spent in A before switching to B* is exponentially distributed with mean $r_{A\to B}^{-1}$: $T_{A\to B} \sim \text{Exp}(r_{A\to B})$. Therefore, we estimated the different rates summarised in the section below by using survival analysis and assuming a exponentially distributed times.

Rates related to movement between CD4 classes
All the rates that are related to the progression of the disease (switch to another CD4 class) within the same care stage are estimated with IeDEA data. As the time spent on one care stage before switching to another stage is generally long, the interval censoring approach provided accurate estimates of almost all rates. As no reliable data exist about untreated individuals (I and D), the progression in CD4 counts for I and D was estimated from literature [11].

Rates related to movement between care stages
Rates that model HIV-transmission, diagnosis and treatment initiation were not estimated with IeDEA data and have already been described in Section 1. For all the other rates that are related to continuum of care, we adapted the survival analysis method to handle the sparsity of IeDEA data. First, as few VL measurements are reported per individuals, some steps are missing in the IeDEA data, e.g. some individuals passing directly from "diagnosed" (D) to "suppressed"

(S_1), which implies that treatment start is missing in the database. In this case, we aimed to reconstruct the history of care of these patients based on the sparse information we had from IeDEA. In this example, as no rate exists between D and S_1, we should assume that the individual stayed at T_1 before going to S_1. Therefore, the information provided by IeDEA data could here help us to estimate two rates: $\gamma_{D\to T_1}$ and $\gamma_{T_1\to S_1}$.
Second, we also modified the method in order to take into account the risk of bias caused by the sparsity of the CD4 measurements. As an example, let's suppose that we want to estimate the suppression rate $\gamma^1_{T_1\to S_1}$ for individuals with $CD4 > 500c/\mu l$. If the CD4 counts fell below $500c/\mu l$ between the last time he has been reported at T_1 and the first time he was at S_1, it is impossible to know whether the patient got suppressed when his CD4 counts was below or above $500c/\mu l$. Therefore, information about this patient cannot be used to inform any of the two rates $\gamma^1_{T_1\to S_1}$ or $\gamma^2_{T_1\to S_1}$. Although the quality of the estimates remains good for most of them due to the high number of individuals, rejecting those patients could introduce a bias. We tried to correct for this bias by adapting the standard method. Let's consider the rate $\gamma^1_{T_1\to S_1}$. First, we estimated this rate without CD4 stratification: $\widehat{\gamma}_{T_1\to S_1}$. Next, we compared the weighted average of the rate stratified by CD4 with its estimate without CD4 stratification, by taking the ratio:

$$c := \frac{\widehat{\gamma}_{T_1\to S_1}}{\left(\sum_{i=1}^{4}\omega_i\cdot\widehat{\gamma}^i_{T_1\to S_1}\right)/\sum\omega_i}. \tag{19}$$

This constant c represents the bias made when estimating $\widehat{\gamma}_{T_1\to S_1}$ by CD4 stratification. To correct for this bias, we therefore multiplied the previously estimated rates $\widehat{\gamma}^i_{T_1\to S_1}$ by c: $c\cdot\widehat{\gamma}^i_{T_1\to S_1}$.
Table 2 shows estimates of the rates that are related to the progression of the disease and Table 3 estimates of the rates that are related to the continuum of care.

Parameter	Description	Values		
		CD4 class		
		$1\to 2$	$2\to 3$	$3\to 4$
$1/\nu^I_{CD4}$	Time before leaving one CD4 class to another one (estimated from [11])	60	36	42
$1/\nu^D_{CD4}$	idem (estimated from [11])	60	36	42
$1/\nu^{T_1}_{CD4}$	idem	28	18	45
$1/\nu^{F_1}_{CD4}$	idem	17	11	16
$1/\nu^{T_2}_{CD4}$	idem	28	18	45
$1/\nu^{F_2}_{CD4}$	idem	15	14	11
		$1\leftarrow 2$	$2\leftarrow 3$	$3\leftarrow 4$
$1/\tilde{\nu}^{T_1}_{CD4}$	idem	13	22	115
$1/\tilde{\nu}^{S_1}_{CD4}$	idem	15	13	10
$1/\tilde{\nu}^{T_2}_{CD4}$	idem (set as $1/\tilde{\nu}^{T_1}_{CD4}$)	13	22	115
$1/\tilde{\nu}^{S_2}_{CD4}$	idem	14	13	8

Table 2: Inverse of the rates (in month) related to disease progression estimated with survival analysis and IeDEA data. Because no or too little data were available to measure them accurately, two rates ($1/\nu^I_{CD4}$ and $1/\nu^{T_2}_{CD4}$) were approximation by other similar rates.

Parameter	Description	Values			
		CD4 class			
		1	2	3	4
$1/\gamma_{T_1 \to S_1}$	Time from T_1 to S_1	6	9	9	14
$1/\gamma_{T_1 \to F_1}$	Time from T_1 to F_1	23	22	15	13
$1/\gamma_{S_1 \to F_1}$	Time from S_1 to F_1	621	460	225	87
$1/\gamma_{F_1 \to S_1}$	Time from F_1 to S_1	28	46	48	53
$1/\gamma_{F_1 \to T_2}$	Time from F_1 to T_2	94	161	65	35
$1/\gamma_{T_2 \to S_2}$	Time from T_2 to S_2	5	8	4	4
$1/\gamma_{T_2 \to F_2}$	Time from T_2 to F_2	20	24	12	8
$1/\gamma_{S_2 \to F_2}$	Time from S_2 to F_2	59	43	40	21
$1/\gamma_{F_2 \to S_2}$	Time from F_2 to S_2	2	10	6	12
$1/\gamma_{T_1 \to D}$	Time from T_1 to D (stop treatment)	414	322	172	156
$1/\gamma_{S_1 \to D}$	Time from S_1 to D (stop treat.)	2069	1241	759	368
$1/\gamma_{F_1 \to D}$	Time from F_1 to D (stop treat.)	621	478	285	129
$1/\gamma_{T_2 \to D}$	Time from T_2 to D (stop treatment)	414	322	172	156
$1/\gamma_{S_2 \to D}$	Time from S_2 to D (stop treat.)	2069	1241	759	368
$1/\gamma_{F_2 \to D}$	Time from F_2 to D (stop treat.)	621	478	285	129
$1/\gamma_{D \to T_2}$	Time from D to T_2	1149	2759	2989	425

Table 3: Inverse of the rates (in month) related to continuum of care estimated with survival analysis and IeDEA data.

3.2 Likelihood maximisation

Four different rates - transmission rate, diagnosis rate, ART initiation rate and mortality rate - were estimated during the second phase. These four rates were modelled with 7 parameters (see Table 5). Estimates from the first phase (Table 2 and 3) and from literature (Table 4) were used to run the model. We used an maximum likelihood approach to fit four model outcomes to outcomes from Thembisa model (see Eq 20). The four model outcomes are: the number of yearly new infection ΔInf, the number of undiagnosed individuals $Diag$, the number of treated individuals $Treat$ and the number of AIDS-related deaths $Mort$.
We used the `optim` function in `R` together with the `L-BFGS-B` method. This method allowed us to provide lower and upper bounds for each parameters. These bounds were chosen in order to include all reasonable values. As optimisation of the likelihood function might give different results depending on the starting values of the parameters, different starting values were randomly chosen within the range defined by the lower and upper bounds. We selected the set of parameters that maximized the likelihood over the simulations that converged. Calculations were performed

on UBELIX (http://www.id.unibe.ch/hpc), the HPC cluster at the University of Bern.

$$\begin{aligned}\log L = &\sum_{i=2005}^{2015} \log \text{Poisson}\left(\Delta Inf_{data}(t), \lambda = \Delta Inf(t)\right) \\ &+ \sum_{i=2005}^{2015} \log \text{Poisson}\left(Diag_{data}(t), \lambda = Diag(t)\right) \\ &+ \sum_{i=2005}^{2015} \log \text{Poisson}\left(Mort_{data}(t), \lambda = Mort(t)\right) \\ &+ \sum_{i=2005}^{2015} \log \text{Poisson}\left(Treat_{data}(t), \lambda = Treat(t)\right)\end{aligned} \tag{20}$$

Parameter	Description	Values	Ref		
Resistance parameters					
$1/\sigma_{res}$	Time to acquire resistance (in month)	5	[12, 13, 14, 15, 16]		
$1/\sigma_{rev}$	Time to revert back to "drug-susceptible" (in month)	120	[17]		
α	Hazard ratio of "being suppressed" between drug-susceptible and drug-resistant individuals	2	[7]		
Other parameters					
$p_{I\to D}$	ratio of diagnosis rate between asymptomatic men and women	0.8			
$\nu_{0,0}$	probability that a male infects a male (per act)	0.8%	[18]		
$\nu_{0,1}$	probability that a male infects a female (per act)	0.3%	[18]		
$\nu_{1,0}$	probability that a female infects a male (per act)	0.3%	[18]		
$\rho_{0,0}$	percentage of MSM	5%	[1]		
μ^i	relative mortality risk		[2] [3]		
	(Ref: suppressed indiv. with CD4>500)	CD4 class			
		1	2	3	4
	$\mu^i_{I/D}$: not treated (I and D)	1.6	2	4.6	40.9-134.4
	$\mu^i_{T_1/T_2}$: started treatment (T_1 and T_2)	2.5	2.6	3.1	10-50.7
	$\mu^i_{S_1/S_2}$: suppressed (S_1 and S_2)	1	1.3	2	8.3-41.7
	$\mu^i_{F_1/F_2}$: failed (F_1 and F_2)	3.9	3.9	4.3	11.8-59.7

Table 4: Parameters collected from literature.

Parameter	Description	Values
β_u	number of unprotected sexual acts per month	3.3
	(for undiagnosed individual)	
β_d	number of unprotected sexual acts per month	1.8
	(for diagnosed individual)	
$\gamma_{I\to D}(2016)/\gamma_{I\to D}(2005)$	Ratio of diagnosis rates between 2005 and 2016	7.7
$1/(12\cdot\gamma_{I\to D}(2005))$	time to diagnosis in 2005	22.8
	in asymptotic HIV-infected men (in year)	
$1/(12\cdot\gamma_{D\to T_1}(2005))$	time to ART initiation in 2005 (in year)	7.8
q	parameter linking the increase of treatment rate with	0.05
	the decrease of the prop. of ind. with $CD4 < 50$ cell/μl	(see Eq 3)
μ_0	Mortality risk (in $(\text{month}\cdot 1000\text{ people})^{-1}$)	0.16
	for a suppressed individual with $CD4 > 500$ cell/μl	

Table 5: Parameters estimated during the second-phase calibration (likelihood maximisation). Rates are in month^{-1}.

4 Results

4.1 Best fits

Figure 2 a,b,c,d shows the 10 best fits among those that converged. Figure 2 e and f shows ADR and TDR levels over time as estimated by the model using the formulas 15 and 16 respectively. The table 6 shows some characteristics of the studies that are used in Figure 2 e and f.

Where	Level	CI (95%)	sample size	Year	Reference
**ADR levels**					
Cape Town	88%	80.3% – 93.3%	110	2002-2007	[12]
8 over the 9 South African provinces	95.4%	93.7% – 96.7%	788	2013-2014	[19]
(no data from Northern Cape province)					
**TDR levels**					
South Africa (meta-analysis)	0% – 10.1%	not given	41 – 1719	2005-2014	[20]
			(total: 5064)		

Table 6: Cross-sectional studies used to compare resistance outcomes of the model (levels of ADR and TDR over time).

4.2 Sensitivity analysis

In the sensitivity analysis, we perturbed 200 times seven parameters using a Latin Hypercube Sampling method (see Table 7). As varying the transmission-related parameters may modify the overall transmission rate, an adjustment is made to have a transmission rate similar to the baseline model. Sensitivity analysis were run for the baseline model as well as for each

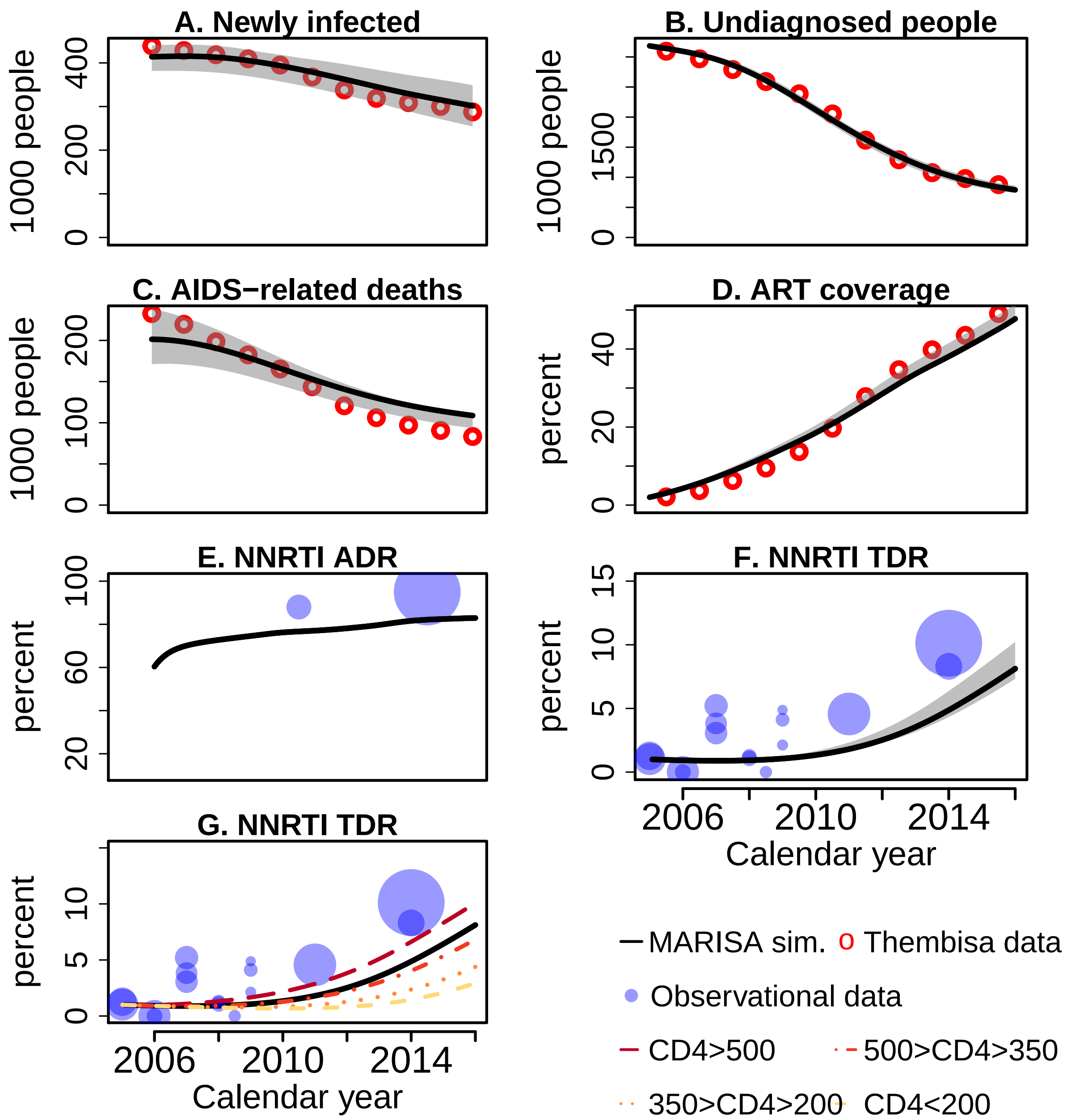

Figure 2: The plots A, B, C and D correspond to the four indicators used during the fitting procedure: A. the number of newly infected per year, B. the number of undiagnosed individuals, C. the number of AIDS-related deaths per year and D. the percentage of infected individuals that are on ART. ADR and TDR levels are displayed in Figure E. and F. respectively. Note, however, that these two latter indicators are not used during the fitting procedure. The lines correspond to the best model fit while the grey area is delimited by the lower and upper bounds of the 10 best fits, whose variation is due to different starting points (see Section 3.2).

counterfactual scenario (10 different scenarios in total). 100% sensitivity ranges are displayed in Figures 2 and 3 and Table 1 of the main paper.

Parameters	Value	Lower Bound	Upper bound
Resistance-related parameters			
$1/\sigma_{res}$	5	3	9
$1/\sigma_{rev}$	125	36	200
α	2	1	5
$\gamma_{T_1\to D}$, $\gamma_{S_1\to D}$, $\gamma_{F_1\to D}$	rate γ (see Table 3)	γ	$2\cdot\gamma$
Transmission-related parameters			
$\rho_{0,0}$	5%	1%	10%
$\nu_{0,0}/\nu_{0,1}$	2.7	1	5
Ratio between HIV prevalence in MSM and in HET	1	1	3

Table 7: Parameter ranges used in sensitivity analysis.

References

[1] Anova Health Institute. Rapid Assessment of HIV Prevention, Care and Treatment Programming for MSM in South Africa; 2013.

[2] Maduna PH, Dolan M, Kondlo L, Mabuza H, Dlamini JN, Polis M, et al. Morbidity and Mortality According to Latest CD4+ Cell Count among HIV Positive Individuals in South Africa Who Enrolled in Project Phidisa. PLOS ONE. 2015 apr;10(4):e0121843. Available from: http://www.ncbi.nlm.nih.gov/pubmed/25856495http://www.pubmedcentral.nih.gov/articlerender.fcgi?artid=PMC4391777http://dx.plos.org/10.1371/journal.pone.0121843.

[3] Brennan AT, Maskew M, Sanne I, Fox MP. The interplay between CD4 cell count, viral load suppression and duration of antiretroviral therapy on mortality in a resource-limited setting. Tropical medicine & international health : TM & IH. 2013 may;18(5):619–31. Available from: http://www.ncbi.nlm.nih.gov/pubmed/23419157http://www.pubmedcentral.nih.gov/articlerender.fcgi?artid=PMC3625450.

[4] Johnson LF, Dorrington RE. Thembisa version 4.1: A model for evaluating the impact of HIV/AIDS in South Africa; 2018.

[5] Boulle A, Bock P, Osler M, Cohen K, Channing L, Hilderbrand K, et al. Antiretroviral therapy and early mortality in South Africa. Bulletin of the World Health Organization. 2008 sep;86(9):678–87. Available from: http://www.ncbi.nlm.nih.gov/pubmed/18797643http://www.pubmedcentral.nih.gov/articlerender.fcgi?artid=PMC2649489.

[6] Johnson LF, Rehle TM, Jooste S, Bekker LG. Rates of HIV testing and diagnosis in South Africa. AIDS. 2015 jul;29(11):1401–1409. Available from: http://www.ncbi.nlm.nih.gov/pubmed/26091299http://content.wkhealth.com/linkback/openurl?sid=WKPTLP:landingpage{&}an=00002030-201507170-00015.

[7] Wittkop L, Günthard HF, de Wolf F, Dunn D, Cozzi-Lepri A, de Luca A, et al. Effect of transmitted drug resistance on virological and immunological response to initial combination antiretroviral therapy for HIV (EuroCoord-CHAIN joint project): a European

multicohort study. The Lancet Infectious Diseases. 2011 may;11(5):363–371. Available from: http://www.ncbi.nlm.nih.gov/pubmed/21354861http://linkinghub.elsevier.com/retrieve/pii/S1473309911700329.

[8] (WHO) WHO. WHO | South Africa; 2018. Available from: http://www.who.int/countries/zaf/en/.

[9] Fairlie L, Bernheimer J, Sipambo N, Fick C, Kuhn L. Lamivudine monotherapy in children and adolescents: The devil is in the detail. South African Medical Journal. 2017 nov;107(12):1055. Available from: http://www.samj.org.za/index.php/samj/article/view/12151.

[10] Muri L, Gamell A, Ntamatungiro AJ, Glass TR, Luwanda LB, Battegay M, et al. Development of HIV drug resistance and therapeutic failure in children and adolescents in rural Tanzania: an emerging public health concern. AIDS. 2016;Available from: https://www.ncbi.nlm.nih.gov/pmc/articles/PMC5131685/pdf/aids-31-061.pdf.

[11] Mangal TD, UNAIDS Working Group on CD4 Progression and Mortality Amongst HIV Seroconverters including the CASCADE Collaboration in EuroCoord. Joint estimation of CD4+ cell progression and survival in untreated individuals with HIV-1 infection. AIDS. 2017 may;31(8):1073–1082. Available from: http://www.ncbi.nlm.nih.gov/pubmed/28301424http://www.pubmedcentral.nih.gov/articlerender.fcgi?artid=PMC5414573http://insights.ovid.com/crossref?an=00002030-201705150-00003.

[12] Orrell C, Walensky RP, Losina E, Pitt J, Freedberg KA, Wood R. HIV type-1 clade C resistance genotypes in treatment-naive patients and after first virological failure in a large community antiretroviral therapy programme. Antiviral therapy. 2009;14(4):523–31. Available from: http://www.ncbi.nlm.nih.gov/pubmed/19578237http://www.pubmedcentral.nih.gov/articlerender.fcgi?artid=PMC3211093.

[13] Sigaloff KCE, Ramatsebe T, Viana R, de Wit TFR, Wallis CL, Stevens WS. Accumulation of HIV Drug Resistance Mutations in Patients Failing First-Line Antiretroviral Treatment in South Africa. AIDS Research and Human Retroviruses. 2012 feb;28(2):171–175. Available from: http://www.liebertpub.com/doi/10.1089/aid.2011.0136.

[14] Wallis CL, Mellors JW, Venter WDF, Sanne I, Stevens W. Varied Patterns of HIV-1 Drug Resistance on Failing First-Line Antiretroviral Therapy in South Africa. JAIDS Journal of Acquired Immune Deficiency Syndromes. 2010 apr;53(4):480–484. Available from: https://insights.ovid.com/crossref?an=00126334-201004010-00007.

[15] Manasa J, Lessells RJ, Skingsley A, Naidu KK, Newell ML, McGrath N, et al. High-Levels of Acquired Drug Resistance in Adult Patients Failing First-Line Antiretroviral Therapy in a Rural HIV Treatment Programme in KwaZulu-Natal, South Africa. PLoS ONE. 2013 aug;8(8):e72152. Available from: https://dx.plos.org/10.1371/journal.pone.0072152.

[16] van Zyl GU, van der Merwe L, Claassen M, Zeier M, Preiser W. Antiretroviral resistance patterns and factors associated with resistance in adult patients failing NNRTI-based regimens in the western cape, South Africa. Journal of Medical Virology. 2011 oct;83(10):1764–1769. Available from: http://doi.wiley.com/10.1002/jmv.22189.

[17] Yang WL, Kouyos RD, Böni J, Yerly S, Klimkait T, Aubert V, et al. Persistence of Transmitted HIV-1 Drug Resistance Mutations Associated with Fitness Costs and Viral Genetic Backgrounds. PLOS Pathogens. 2015 mar;11(3):e1004722. Available from: http://dx.plos.org/10.1371/journal.ppat.1004722.

[18] Patel P, Borkowf CB, Brooks JT, Lasry A, Lansky A, Mermin J. Estimating per-act HIV transmission risk. AIDS. 2014 jun;28(10):1509–1519. Available from: http://www.ncbi.nlm.nih.gov/pubmed/24809629http://content.wkhealth.com/linkback/openurl?sid=WKPTLP:landingpage{&}an=00002030-201406190-00014.

[19] Steegen K, Bronze M, Papathanasopoulos MA, van Zyl G, Goedhals D, Variava E, et al. HIV-1 antiretroviral drug resistance patterns in patients failing NNRTI-based treatment: results from a national survey in South Africa. Journal of Antimicrobial Chemotherapy. 2017 jan;72(1):210–219. Available from: http://www.ncbi.nlm.nih.gov/pubmed/27659733https://academic.oup.com/jac/article-lookup/doi/10.1093/jac/dkw358.

[20] Gupta RK, Gregson J, Parkin N, Haile-Selassie H, Tanuri A, Andrade Forero L, et al. HIV-1 drug resistance before initiation or re-initiation of first-line antiretroviral therapy in low-income and middle-income countries: a systematic review and meta-regression analysis. The Lancet Infectious diseases. 2018 mar;18(3):346–355. Available from: http://www.ncbi.nlm.nih.gov/pubmed/29198909http://www.pubmedcentral.nih.gov/articlerender.fcgi?artid=PMC5835664.
